# Characterization of the *cis* elements in the proximal promoter regions of the anthocyanin pathway genes reveals a common regulatory logic that governs pathway regulation

**DOI:** 10.1093/jxb/erv173

**Published:** 2015-04-23

**Authors:** Zhixin Zhu, Hailong Wang, Yiting Wang, Shan Guan, Fang Wang, Jingyu Tang, Ruijuan Zhang, Lulu Xie, Yingqing Lu

**Affiliations:** ^1^State Key Laboratory of Systematic and Evolutionary Botany, Institute of Botany, Chinese Academy of Sciences, 20 Nan Xin Cun, Beijing 100093, China; ^2^University of Chinese Academy of Sciences, Beijing 100049, China

**Keywords:** Anthocyanin pathway, *Arabidopsis*, bHLH, *cis* element, *Ipomoea*, MBW complex, MYB, promoter activity, promoter architecture, regulatory module, *trans* factor, transcription initiation, WDR.

## Abstract

Cellular activities such as compound synthesis often require the transcriptional activation of an entire pathway; however, the molecular mechanisms underlying pathway activation have rarely been explained. Here, the *cis* regulatory architecture of the anthocyanin pathway genes targeted by the transcription factor (TF) complex including MYB, bHLH, and WDR was systematically analysed in one species and the findings extended to others. In *Ipomoea purpurea*, the IpMYB1-IpbHLH2-IpWDR1 (IpMBW) complex was found to be orthologous to the PAP1-GL3-TTG1 (AtPGT) complex of *Arabidopsis thaliana*, and interacted with a 7-bp MYB-recognizing element (MRE) and a 6-bp bHLH-recognizing element (BRE) at the proximal promoter region of the pathway genes. There was little transcription of the gene in the absence of the MRE or BRE. The *cis* elements identified experimentally converged on two syntaxes, ANCNNCC for MREs and CACN(A/C/T)(G/T) for BREs, and our bioinformatic analysis showed that these were present within anthocyanin gene promoters in at least 35 species, including both gymnosperms and angiosperms. For the anthocyanin pathway, IpMBW and AtPGT recognized the interspecific promoters of both early and later genes. In *A. thaliana*, the seed-specific TF complex (TT2, TT8, and TTG1) may regulate all the anthocyanin pathway genes, in addition to the proanthocyanidin-specific *BAN*. When multiple TF complexes in the anthocyanin pathway were compared, the *cis* architecture played a role larger than the TF complex in determining the variation in promoter activity. Collectively, a *cis* logic common to the pathway gene promoters was found, and this logic is essential for the *trans* factors to regulate the pathway.

## Highlight

MYB and bHLH bind to two identifiable syntaxes present in target gene promoters, and the anthocyanin pathway is regulated as one unit as in other branches of the flavonoid network.

## Introduction

Anthocyanins are widely synthesized in seed plants to provide colouration, protection under various circumstances, and components for cellular activities. The synthesis of anthocyanins is the end product of genetically well characterized enzymes (Winkel-Shirley, 2001). These enzymes also comprise the backbone of the flavonoid synthesis network, supporting the network to metabolize arrays of secondary compounds in different species (Vogt, 2010). Although the regulation of the anthocyanin pathway genes has been known to be under the control of a transcription factor (TF) complex that consists of MYB, bHLH, and WD-repeat (WDR) proteins (Broun, 2004; Koes *et al.*, 2005; Ramsay and Glover, 2005; Xu *et al.*, 2015), little consensus has been reached about the *cis* elements recognized by the complex at the pathway level. The MYB-bHLH-WDR (MBW) complex represents a mode of gene regulation not only for the anthocyanin pathway but also proanthocyanidin (PA) synthesis and epidermal cell differentiation (reviewed by Feller *et al.*, 2011). Systematic examination of the *cis* structures involved in the regulation of pathway genes is important for understanding these biological processes.

The involvement of MYB and bHLH in flavonoid gene regulation was initially found in *Zea mays*, with mutants of *c1* (Paz-Ares et al., 1987) and *Lc* (Ludwig *et al.*, 1989), respectively; the necessity for both MYB and bHLH in the activation of the anthocyanin genes was soon established in this species (Goff *et al.*, 1990; Bodeau and Walbot, 1992). Still in maize, details followed that the N-terminus of B (a bHLH) directly interacted with the R2R3 domain MYB C1 (Goff *et al.*, 1992), and that the C-terminus of C1 was responsible for transcriptional activation (Sainz *et al.*, 1997). Interestingly, *C1* and *Lc* were both required for restoring the phenotype of *an11* mutant in *Petunia* (Quattrocchio *et al.*, 1993), and *AN11* was later found to be a WDR regulating the anthocyanin pathway (deVetten *et al.*, 1997). The participation of WDR in the pathway was further validated with *Arabidopsis* TTG1 (Walker *et al.*, 1999) and *Zea* PAC1 (Carey *et al.*, 2004).

In *Arabidopsis*, characterization of MBWs led to the identification of PAP1-GL3/EGL3-TTG1 transiently expressed in the seedlings for anthocyanin synthesis (Zhang *et al.*, 2003; Gonzalez *et al.*, 2008) and TT2-TT8-TTG1 in developing siliques for PA synthesis (Nesi *et al.*, 2001; Baudry *et al.*, 2004; Lepiniec *et al.*, 2006). At the same time, anthocyanin regulatory genes *MYB1*, *bHLH2*, and *WDR1* were reported from *Ipomoea* mutants (Chang *et al.*, 2005; Morita *et al.*, 2006; Park *et al.*, 2007), complementing previously well characterized *AN2*-*AN1*-*AN11* in the *Petunia* hybrid Vilmorin (Beld *et al.*, 1989; Quattrocchio *et al.*, 1993; deVetten *et al.*, 1997). Nonetheless, the collaborative action of the *Ipomoea* TFs requires more evidence. Reported components of the anthocyanin MBW complexes appear to form their own clades, with MYBs from the subgroup 6 (Dubos *et al.*, 2010), bHLHs (subfamily IIIf) (Pires and Dolan, 2010), and recently WDRs (subgroup 19) (Li *et al.*, 2014).

Binding of MYB and bHLH to promoters of anthocyanin structural genes was first reported with maize C1 and B (Roth *et al.*, 1991). Details showed that from –123 to –88bp of the *A1* promoter was critical for C1/B activation (Tuerck and Fromm, 1994); a region within –224bp of the *Bz2* promoter was adequately regulated by R (a B homologue) and C1 (Bodeau and Walbot, 1996). For the MYB part, C1 could bind to variable sites in the maize *a1* gene promoter (Sainz *et al.*, 1997), and a 16 bp-long consensus motif was identified from *a2* and other C1-binding genes (Lesnick and Chandler, 1998); recently, the consensus was narrowed down to ANCNACC by site mutagenesis tests with anthocyanin MYB1s in coloured *Ipomoea* petals and magnolia tepels (Wang *et al.*, 2013). For the bHLH part, after reported binding of CG-1 protein (Staiger *et al.*, 1991) and human c-MYC (Blackwell *et al.*, 1990) to CACGTG, the G-box was recently shown to bind to maize R (Kong *et al.*, 2012), petunia AN1, and *Ipomoea* bHLH2 (Wang *et al.*, 2013). EMSA tests on anthocyanin gene promoter fragments involving *Ipomoea CHS-D*, *Zea 3GT*, and *Gerbera DFR* led to a tentative consensus of bHLH-recognized elements in the form of CACNN(G/T) (Wang *et al.*, 2013). All these analyses agreed in that the binding of MYB and bHLH occurred at locations within 200bp upstream of the translation start site. This feature of *cis* locations was also reported in bean (Loake *et al.*, 1992), grape (Gollop *et al.*, 2002), African daisy (Elomaa *et al.*, 2003), and apple (Espley *et al.*, 2009), indicating that the short functional promoters are common to the anthocyanin genes. The focus of these studies was usually on one or a few promoters. Analysis of TF–promoter interactions at the level of pathway has been limited to several *Arabidopsis* promoters activated by maize TFs (Hartmann *et al.*, 2005). How conspecific pathway genes interact in the 5′-noncoding region remains to be explored. Obviously, conspecific TF interactions are most relevant to understanding pathway regulation.

In documented MBW complexes, specific residues were identified on maize C1 for its interaction with R (Grotewold *et al.*, 2000), while protein interaction was also observed between *Arabidopsis* GL3 and TTG1 (Payne *et al.*, 2000); however, little interaction was observed between MYB and WDR (Zhang *et al.*, 2003). Meanwhile, transcription of *Arabidopsis TT8* and petunia *AN1* require the presence of both MYB and WDR (Baudry *et al.*, 2006; Albert *et al.*, 2014). For transcription of MYB and WDR, however, the necessity for the presence of MBW remains unclear, except in one case of a MYB mutant (Espley *et al.*, 2009). With all that is known about MBW regulation, however, little can be said about the conspecific interactions between the *trans* and *cis* components. Although anthocyanin TFs have been found to be conserved in both monocots and dicots (e.g. Quattrocchio *et al.*, 1993; Hartmann *et al.*, 2005), the molecular mechanism for observed *trans*-specific regulation is unclear. While the lack of identified *cis* components and the range of a gene’s promoter previously prevented systematic and quantitative analysis of the regulatory mechanism, accumulating data indicate that transcription in metazoa typically occurs within the proximal promoter region (Lenhard *et al.*, 2012), which appears true also for the anthocyanin genes. With a recently reported method (Wang *et al.*, 2013), the *cis* element(s) may be pinpointed to an anthocyanin promoter through both bioinformatic and experimental approaches, and quantitative evaluation of contributions of TFs and *cis* elements to gene regulation is now achievable.

In order to elucidate the molecular mechanism(s) of anthocyanin pathway regulation in the proximal regions, we analysed interactions of TFs and *cis* motifs of conspecific genes at the pathway level in *I. purpurea* Roth (common morning glory) and made parallel examinations of the homologous genes in *Arabidopsis thaliana* (L.) Heynh. as a reference. The *I. purpurea* genes included those encoding chalcone synthase D (*CHS-D*), chalcone isomerase (*CHI*), flavanone 3-hydroxylase (*F3H*), flavonoid 3′-hydroxylase (*F3′H*), dihydroflavonol reductase B (*DFR-B*), anthocyanidin synthase (*ANS*), UDP-glucose:flavonoid 3-*O*-glucosyltransferase (*3GT*), UDP-glucose:anthocyanidin 3-*O*-glucoside-2′-*O*-glucosyltransferase (*3GGT*), and the *trans* factors MYB1, bHLH2, and WDR1. Transcriptional activities of MBW complexes were subsequently compared between *I. purpurea* and *Arabidopsis* on interspecific and conspecific promoters. A bioinformatic analysis of homologues in multiple species was used to explore the generality of *cis* patterns (initially identified in *I. purpurea*). These extensive analyses revealed the features of the MYB- and bHLH- recognizing elements (MREs and BREs, respectively), patterns of MREs and BREs on the proximal promoters, and one *cis* logic for pathway regulation.

## Materials and methods

### Plant materials

Seeds of *I. purpurea* taken from a nationwide collection (Lu *et al.*, 2009) were grown in the botanical garden of IB-CAS, Beijing. These plants provided petal tissue for anthocyanin gene cloning and quantitative analysis of gene expression, and leaf tissue for promoter amplification and genotyping. Seeds of *Arabidopsis* (*tt3* mutant and the Columbia accession) were obtained from the *Arabidopsis* information resource (TAIR) and those of *Petunia hybrida* (R27) were a kind gift from Dr Quattrocchio (Free University, The Netherlands). All seeds were germinated in the growth chamber.

### qRT-PCRs

RNA samples were extracted in TRIzol (Life Technologies) from petals of *I. purpurea* (III6-4 and III6-9). Each plant was sampled at 2-h intervals starting 60h before flower opening. RNAs were reverse transcribed and quantifications of transcript copies followed the protocol reported by Lu *et al.* (2012). About 1–10ng cDNA was used in each qPCR reaction, and the running conditions were optimized for efficiency with each gene taken (Supplementary Table S1).

### Gene cloning and sequencing

Standard PCRs were applied to leaf genomic DNAs to amplify five gene promoters (*IpCHS-D*, *IpF3′H*, *IpDFR-B*, *IpbHLH2*, and *IpWDR1*) based on primers (Supplementary Table S1) whose designs followed the available sequences of conspecific or closely related targets (Supplementary Table S2). The promoter sequence of *IpF3H* was obtained by inverse PCR (Ochman *et al.*, 1988). Promoter sequences of *IpMYB1*, *IpCHI*, *IpANS*, *Ip3GT*, and *Ip3GGT* were isolated by tail-PCRs (Liu and Whittier, 1995). Homologue promoters from *Arabidopsis* were PCR-amplified via primers targeting the Columbia accession (Supplementary Table S1). *Ipomoea* TFs were reverse transcribed from RNAs extracted from fresh petals of *I. purpurea*. *Arabidopsis* TFs were similarly amplified from seedlings of the Columbia accession with the appropriate primers (Supplementary Table S1).

### Dual luciferase assays

The promoters were ligated into vectors to prepare for dual luciferase assays as described by Wang *et al.* (2013). The complete coding regions of TFs were integrated into pJIT163 (Guerineau *et al.*, 1992) in frame with appropriate restriction sites (Supplementary Table S1) to replace its reporter gene, resulting in effector vectors. The reporter vectors were adapted from pJIT163-eGFP with the CaMV 35S promoters replaced by the proximal promoters and GFP replaced by the firefly luciferase (LUC) gene. The reference vector was also based on pJIT163-eGFP, with its GFP replaced by the renilla luciferase (RUC) gene (Wang *et al.*, 2013). The vectors were introduced into leaf cells of the *Ipomoea nil wdr1* mutant (seeds available on request) by particle bombardment (BioRad PDS-1000/He equipment). A construct mixture (0.5 μg effector, 0.5 μg reporter, and 0.1 μg reference for each shot) was prepared for the plasmid cocktail, which was coated with 50mg ml^–1^ microparticles (Bio-Rad) at 2.0 μl per 1.0 μg plasmid DNA. The complex was then mixed with 2.5M CaCl_2_ and 0.1M spermidine in a ratio of 1:5:2. Other details followed Wang *et al.* (2013).

### Yeast two-hybrid assays

The coding sequences of *IpMYB1*, *IpbHLH2*, and *IpWDR1* were integrated in frame into vectors pAD-GAL4 2.1 and pBD-GAL4 Cam of HybriZAP-2.1 (Stratagene), respectively, according to the manufacturer’s instructions. Constructs were introduced in pairs into the YRG-2 yeast strain (Stratagene). The transformants were plated onto SD media minus histidine, leucine, and tryptophan for identifying positive interactions. The strengths of TF interactions were then measured by *β*-galactosidase activities according to the protocol of the Matchmaker system (Clontech).

### Electrophoretic mobility shift assays

A complete coding sequence of MYB was integrated in frame into pMAL-c2G (New England Biolabs) to express a tagged protein consisting of maltose-binding protein (MBP) and MYB. A partial coding region of bHLH was similarly expressed. The vector-transformed *Escherichia coli* strain BL21 (DE3) was incubated at 37°C in LB solution and treated as described (Wang *et al.*, 2013). Protein quantification followed the Bradford method using the bovine gamma globulin (BIO-RAD) as the standard. Probes were quantified by the Picogreen quantification module of Rotor Gene 3000 (Corbett Research) with λDNA (Life technologies) as the reference. Details for binding reactions and gel documentation followed Wang *et al.* (2013).

### Transgenic experiments

Homozygous *Arabidopsis tt3* mutants were grown in the growth chamber for transgenic tests of the *IpDFR-B* promoter series. The coding regions of *IpDFR-B* were first subcloned into the SN1301 binary vector for the construction of SN1301-35S::DFR-B_coding_. Varying promoter regions (*IpDFRB-1559*, *IpDFRB-191*, and *IpDFRB-166*) independently replaced the 35S promoter to generate series of SN130-proDFR::DFR-B_coding_ vectors. These vectors were singly introduced into *Agrobacterium* strain GV3101 via the standard freeze-thaw protocol. Transformation of *Arabidopsis* followed a modified flower dip protocol (Logemann *et al.*, 2006), and the selection of transformants was on MS medium containing 50mg l^–1^ hygromycin and 50mg l^–1^ carbenicillin. DNAs were subsequently extracted from these plants and scored for the presence of the transgene by PCRs and DNA sequencing.

Phenotypic complementation was observed in the T2 generation. Seeds were sterilized and cultivated on 1/2MS medium containing 5% sucrose, kept at 4°C in darkness for 2 days, and transferred to constant light conditions (100 μmol m^–2^ s^–1^, 24°C) for observations. For 4–5-week old seedlings, treatment by dehydration (up to a week) and a low temperature (10–16°C) also induced anthocyanin accumulation. Anthocyanins extracted from representative seedlings were further examined by high performance liquid chromatography using cyanidin-3-*O*-glucoside (Extrasynthese) as the standard.

### Bioinformatic analysis

Multiple blastp searches in the NCBI database (ftp://ftp.ncbi.nih.gov/genbank/, up to 10 October 2012) identified protein homologues for each of the anthocyanin genes. The results were trimmed for redundancy following the protocol reported by Wang *et al.* (2013). For each of the promoters, a sliding window (6-bp width) was initially adapted to search for four or more bases matching CACNNG; if this condition was met, the second sliding window of 7bp was taken to examine whether or not there were four or more bases matching ANCNNCC in the next 100 bases. When both searches returned hits, the non-coding sequences were recorded and the candidate *cis* elements were uppercased by WxW_Align.gene, and the frequency of each case was counted by WxW_Align.PosD (Supplementary File 1). We ended up with 159 sequences satisfying the search criteria, which provided the basis for mapping the natural distribution of known *cis* elements on anthocyanin gene promoters. The sequences included in the peak of the distribution were further characterized by two motifs identified via MEME software following the parameters of Wang *et al.* (2013).

### Statistical analysis

Standard *t*-tests were performed on comparisons between averages of experiments. Two-way ANOVAs were carried out after log transformation of the promoter activity. Pearson correlation coefficients were calculated with the significance levels corrected for multiple comparisons by the Dunn-Šidák method (taking the experiment-wise error rate *α* = 0.05). All tests were computed using SAS (ver. 9.0).

## Results

### IpMYB1, IpbHLH2, and IpWDR1 collaboratively regulate the anthocyanin pathway genes

Although the regulatory genes *IpMYB1*, *IpbHLH2*, and *IpWDR1* are expressed in the corolla of *I. purpurea* (Chang *et al.*, 2005; Morita *et al.*, 2006; Park *et al.*, 2007; Guan and Lu, 2013), whether or not they regulate the anthocyanin pathway as a complex remains to be confirmed. If they do, expression of the anthocyanin pathway genes is expected to be correlated. We quantified the pair-wise Pearson correlation coefficients among transcript levels of ten anthocyanin genes via reversely transcribed quantitative PCRs (RT-qPCRs) in developing petals of two wild-type *I*. *purpurea* individuals (Supplementary Table S3). The transcript levels of the enzyme-coding (structural) genes (*IpCHS-D*, *IpCHI*, *IpF3H*, *IpDFR-B*, and *IpANS*) were significantly correlated with those of regulatory genes (*IpMYB1*, *IpbHLH2*, and *IpWDR1*) and among themselves (except between *IpDFR-B* and *Ip3GT*). The expression of *IpF3′H* was highly correlated with that of *IpbHLH2* and *IpWDR1,* but not with that of *IpMYB1*. The quantitative data largely agreed with the perception that *IpMYB1*, *IpbHLH2*, and *IpWDR1* regulated pathway transcription collaboratively. The features of the TFs were examined in two further experiments. One showed the subcellular localization of the three TFs. The vectors with fluorescent-tagged TFs were introduced into the onion epidermis (Supplementary Figure S1A), and the *in vivo* images indicated that IpbHLH2 and IpWDR1 were localized in both the nucleus and the cytoplasm, whereas IpMYB1 was mostly in the nucleus. This pattern agrees with the documented subcellular sites of their homologues (Ness *et al.*, 1989; Matus *et al.*, 2010). The other experiment described protein interactions via yeast two-hybrids. Quantifications of β-galactosidase activities suggested significant interactions between IpMYB1 and IpbHLH2 as well as between IpbHLH2 and IpWDR1 (Supplementary Figure S1B), as expected.

**Fig. 1.**
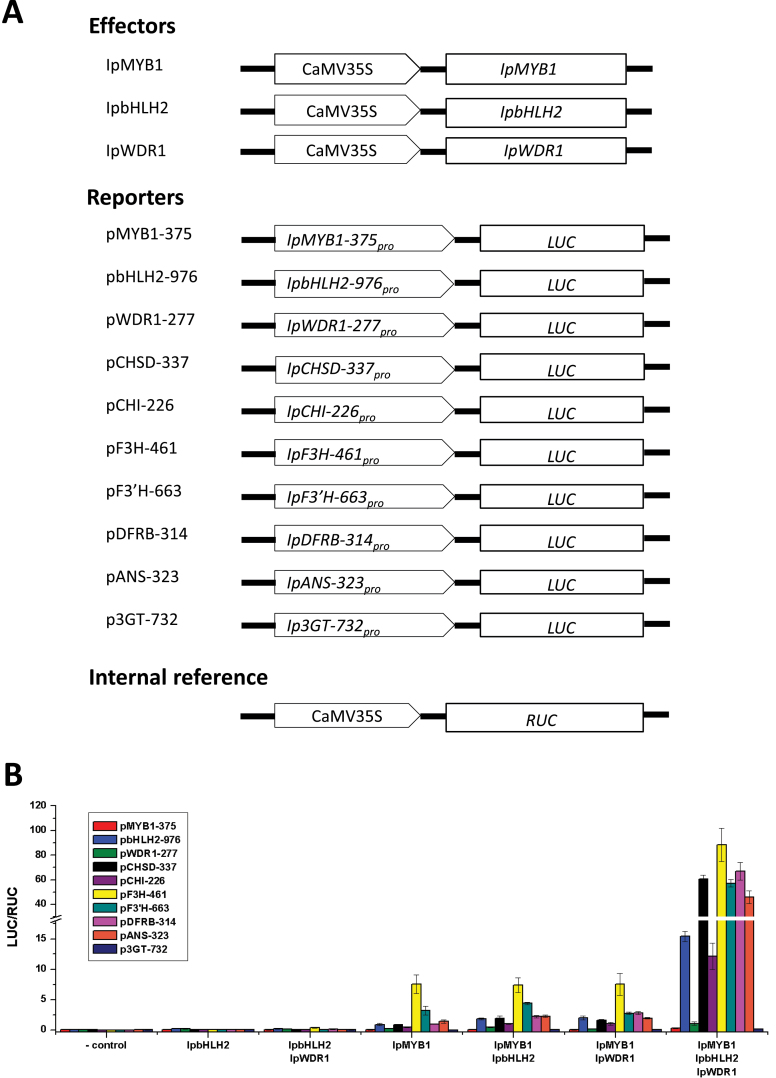
Collaborative regulation of IpMYB1, IpbHLH2, and IpWDR1 on the conspecific genes of the anthocyanin pathway of *I. purpurea*. (A) Construct details for dual luciferase assays. Effectors and reporters were based on the same plasmid pJIT163 (black line). The promoter region of each construct is shown by an arrowed rectangle, while the coding region is shown by the plain rectangle. The firefly luciferase (LUC) was taken as the reporter gene while renilla luciferase (RUC) was the reference for internal control. (B) Results of transient expression assays expressed as the ratios of LUC and RUC for trials on different combinations of TFs. The 5′-noncoding regions are shown using bars for the ten genes tested. The negative controls were the reporters only. Effectors were added as shown on the *x*-axis for each reporter, with the error bars based on four replicates. This figure is available in colour at *JXB* online.

The TF validations paved the way for identification of the conspecific structural genes of the pathway. For *I. purpurea*, some pathway genes (*IpCHS-D*, *IpF3′H*, and *IpDFR-B*) were characterized through mutants or mutant complementation (Habu *et al.*, 1998; Hoshino *et al.*, 2003; Zufall and Rausher, 2004), while others, including *IpCHI*, *IpF3H*, *IpANS*, and *Ip3GT*, were largely identified via gene expression and sequence homology to genes identified in other model species (Tiffin *et al.*, 1998; Durbin *et al.*, 2000; Clegg and Durbin, 2003; Lu *et al.*, 2009). Assuming that they were the *bona fide* genes of the anthocyanin pathway, we collected their 5′-noncoding sequences upstream of the translation start sites (Supplementary Table S2); similar information was also gathered for *IpMYB1*, *IpbHLH2*, and *IpWDR1* to evaluate the TF regulatory capacity on the expression of each. By single and combinatory tests of the TFs as effectors in dual luciferase assays (Fig. 1A), we detected how IpMYB1, IpbHLH2, and IpWDR1 (IpMBW) regulated the reporter gene via the 5′-noncoding regions of the pathway genes. We found that seven (*IpCHS-D*, *IpCHI*, *IpF3H*, *IpF3′H*, *IpDFR-B*, *IpANS*, and *IpbHLH2*) of the ten promoter constructs could be activated strongly by IpMBW; however, the constructs hosting the *IpWDR1* 5′-noncoding regions responded only at low levels to IpMBW, and little promoter activity was found on those hosting various lengths of *Ip3GT* and *IpMYB1* promoter sequences (Supplementary Figure S1C). The tests also showed that IpMYB1 could, alone, initiate a low level of transcription on the seven promoters (particularly *F3H*). By contrast, IpbHLH2 or IpWDR1 alone failed to activate observable promoter activities in the same setting. Only the collective presence of all three TF effectors generated considerable promoter activities (Fig. 1B), suggesting that the TFs acted as an IpMBW complex in transcriptional activation of the main anthocyanin pathway genes in *I. purpurea*. The activation of IpMBW on the promoter of *IpbHLH2* resembles that of *TT8* in *Arabidopsis* (Baudry *et al.*, 2006).

### Two syntaxes emerge from identification of *cis* elements in pathways

**Fig. 2.**
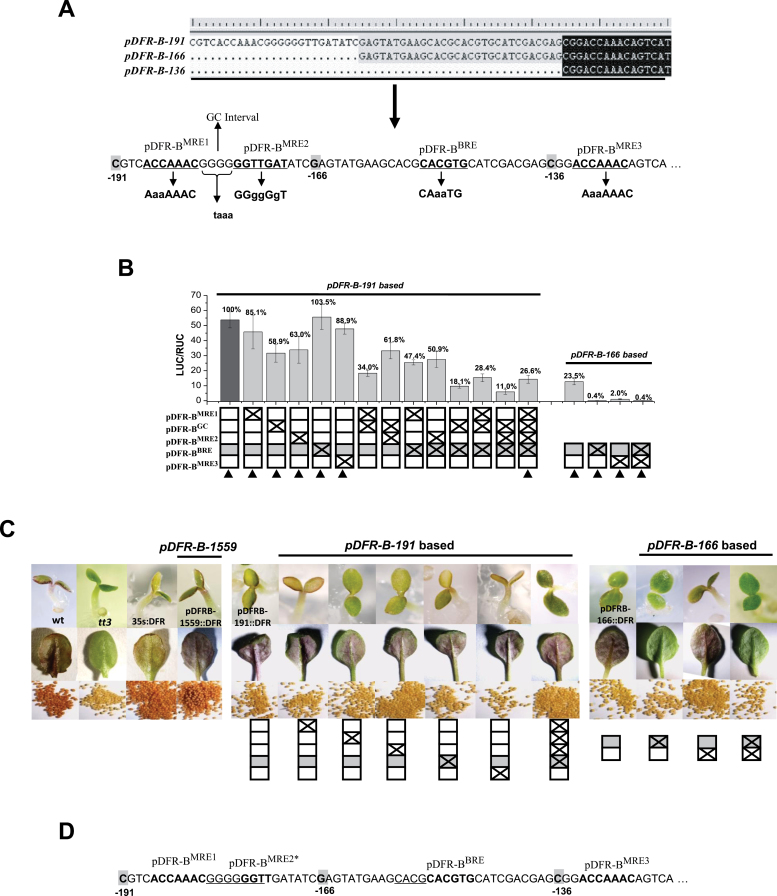
Analysis of *cis* elements on *IpDFR-B*_*pro*_.(A) Sequence features of *pDFR-B-191*, *pDFR-B-166*, and *pDFR-B-136.* Predicted *cis* motifs on *pDFR-B-191*are in bold and underlined, and the mutated sites shown in lower case. The nucleotides that are shaded and in bold mark the beginnings of *pDFR-B-191*, *pDFR-B-166*, and *pDFR-B-136.* (B) Results of dual luciferase assays. Effectors were IpMYB1, IpbHLH2, and IpWDR1 (0.5 µg each). The black bar for *pDFRB-191* was set as 100%. Mutation types (BRE in grey) are checked boxes. The error bars indicate the standard errors based on four independent trials. The tests highlighted by triangles were further examined by the following transgenic trials. (C) Phenotypes of wild type, *dfr* mutant (*tt3*), and transgenic seedlings of *Arabidopsis.* The upper panel shows 2–3 day old seedlings. Some mature leaves were induced by 10–18°C and dehydration. The seeds of each line are shown in the lower panel. The schematic boxes follow the same notation as in (B). (D) A summary of *cis* motifs on *IpDFR-B-191*_*pro*_. The *cis* elements BRE and MRE3 were confirmed on *IpDFR-B-166*_*pro*_ in the mutation tests of transient expression shown in (B) and the additional transgenic *in vivo* test of BRE shown in (C). MRE1 and MRE3 are identical in sequence and weak in effect as seen in (B). Effects of the previous MRE2 and GC interval in (B) were similar whether alone or in combination, and are thus best explained by a unified MRE2*, which was also supported in the subsequent EMSA test with the *IpDFRB-m4* probe. The underlined motifs are possibe additional targets of the TFs *in vivo*. This figure is available in colour at *JXB* online.

**Fig. 3.**
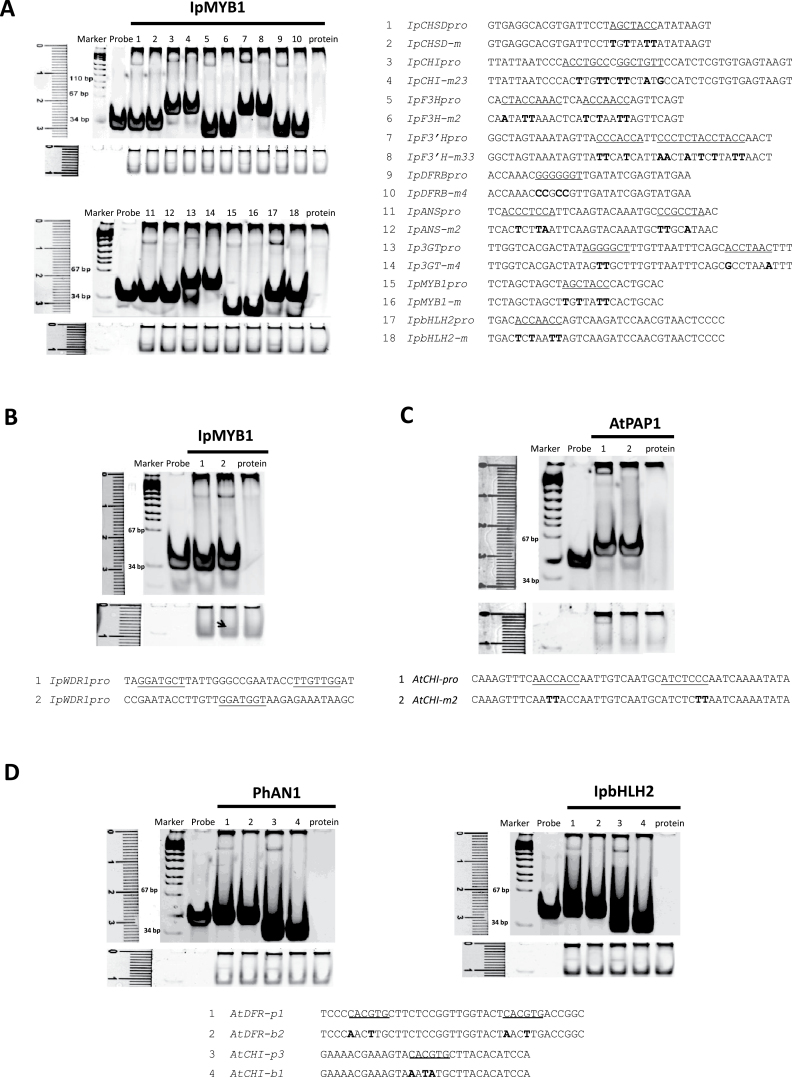
Results of EMSAs with MYBs and bHLHs. (A) *I. purpurea* probes based on the 5′-noncoding regions of the anthocyanin genes and their binding results with IpMYB1 in EMSAs. The probes harboured candidate *cis* motifs (underlined) and their mutations (in bold). The gels are shown in two parts, with the upper panel for the DNA binding and the lower panel for protein binding of the same gel photographed under a different light filter (Wang *et al.*, 2013). The lane numbers correspond to those of probes used in the binding reactions; the probe lane contains probe 1 (20 pmol) only; the protein lane has MBP-IpMYB1 (2 µg) only. (B) Results of EMSAs with probes based on the 5′-noncoding region of *IpWDR1*. Three likely *cis* motifs (underlined) were tested with IpMYB1; only the second probe showed positive binding as indicated by the arrow. The arrangement shown follows that described in (A). (C) *Arabidopsis* probes based on *AtCHI*_*pro*_ and their binding results with AtPAP1. The probes and EMSA results are shown as in (A). (D) EMSAs of bHLHs of *I. purpurea* (IpbHLH2) and *Petunia* (PhAN1) with probes based on the 5′-noncoding regions of *Arabidopsis DFR* and *CHI* genes. The experimental conditions followed (A), and the mutations to the G-box are indicated in bold.

The central aim of our study was to understand how the TFs interacted with the *cis* elements at the target gene promoters. Our identification of *cis* elements was extended from *IpCHS-D*_*pro*_ (Wang *et al.*, 2013) to all other gene promoters including *IpCHI*_*pro*_, *IpF3H*_*pro*_, *IpF3′H*_*pro*_, *IpDFR-B*_*pro*_, *IpANS*_*pro*_, *Ip3GGT*_*pro*_, *IpWDR1*_*pro*_, and *IpbHLH2*_*pro*_. Both sequential deletion tests (Supplementary Figure S1C) on the cloned 5′-noncoding regions of the anthocyanin genes and site-by-site tests on the predicted motifs (concentrated on the proximal promoter regions) were adapted. In the initial case of *IpDFR-B*, three experimental approaches were taken for site-specific analysis, including dual luciferase assays performed on leaves of the *wdr1* mutant of *I. nil,* gene transformations of the *tt3* mutant of *Arabidopsis*, and electrophoretic mobility shift assays (EMSAs) with IpMYB1 and IpbHLH2. Starting by mutating predicted motifs (Fig. 2A), we examined the effects of the mutations in both dual luciferase assays (Fig. 2B) and transgenic plants (Fig. 2C). The presence of multiple MREs was evident for *IpDFR-B-191*_pro_ as mutations at one of the MREs did not much affect the transcriptional intensity, and only mutations at multiple motifs led to a significant reduction in promoter activity (Fig. 2B). The initially suspected effect of the ‘GC interval’ overlapped with that of pDFR-B^MRE2^, and the two were better explained by the modified pDFR-B^MRE2*^ (Fig. 2D). The function of pDFR-B^MRE2*^ was further supported by the EMSA assay with the probe (*DFRBpro*) hosting a suspected motif (GGGGGTT or GGGGGGT, a reverse complement of MRE) as mutations at the site (*DFRB-M4*) demolished the binding capacity of IpMYB1 (Fig. 3A). In the transgenic results, promoter activity as little as 2% could still result in anthocyanin accumulation in the seedlings of *Arabidopsis* (e.g. *pDFR-B-166* ^MRE3^; Fig. 2C); the promoter construct *IpDFRB-1559*_*pro*_ *::IpDFRB-coding* caused a higher anthocyanin accumulation than that of *35S::DFRB-coding* in the *tt3* background (Fig. 2C), suggesting that the 35S promoter did not lead to excessive gene expression *in vivo*. This was relevant to the dual luciferase assay where the effectors were driven by 35S promoters.

Though effective in validating *cis* elements, the time commitment for the transgenic tests and common occurrence of multiple *cis* elements prevented the transgenic approach being used on a large scale. We identified *cis* elements for other anthocyanin genes mainly through mutation tests by means of EMSAs and transient expressions. Locating and testing *cis* elements on those genes were guided partially by the previous results on MREs and BREs (Wang *et al.*, 2013) and partially by our ongoing accumulating data. The EMSA results were presented in both DNA- and protein-staining to reduce false positives. Effective *cis*-containing probes that showed positive binding to MBP-tagged IpMYB1 were identified for all tested anthocyanin genes including *IpCHS-D*, *IpCHI*, *IpF3H*, *IpF3′H*, *IpANS*, *Ip3GT*, *IpMYB1*, *IpbHLH2*, and *IpWDR1* (Fig. 3A, B). To know whether or not TF orthologues could recognize similar *cis* elements, we expressed MBP-tagged PAP1 of *Arabidopsis* to detect its binding capacity to the predicted *cis* elements of *AtCHI*_*pro*_. The results suggested that the MYB orthologues recognized the *cis* elements of similar structure (Fig. 3C). Likewise, IpbHLH2 shared the same recognition pattern with PhAN1 for the G-box present at the promoters of *AtDFR* and *AtCHI* (Fig. 3D), as with the known binding capacity of AtGL3 to the G-box (Shangguan *et al.*, 2008). Further EMSA tests with IpbHLH2 indicated that its BREs could change from canonical CACGTG to CACGTT (Supplementary Figure S2).

Complementing the EMSAs but in a cellular environment, dual luciferase assays showed that mutations correctly targeting MREs and BREs could reduce promoter activities (Supplementary Figure S3). Except in one case (*IpbHLH2*_*pro*_), where accidental mutations to pbHLH^MRE2^ caused an exceedingly high promoter activity, all other introduced mutations were mostly on targets, judging from their reduced promoter activities. To make sure that the TFs were not overexpressed to bias the detection of effective motifs, we compared 10-fold levels of particle bombardment in the assays for two genes including *IpCHSD*_*pro*_ and *IpF3′H*_*pro*_ (Supplementary Figure S4A, B). Although lowered TF dosages led to reduced promoter activities (Supplementary Figure S4C), the patterns of the relative effects of mutated motifs were largely unchanged (Supplementary Figure S4D). To further make sure that the results of mutation tests were not biased by using leaves of *I. purpurea*, we compared the same promoters and effectors in the leaves of cultivated rice seedlings in transient assays. The results showed essentially the same patterns for the mutation effects (Supplementary Figure S4E, F) despite change in promoter strength. These experiments effectively validated applications of transient assays to assessing mutation effect of *cis* motifs.

**Fig. 4.**
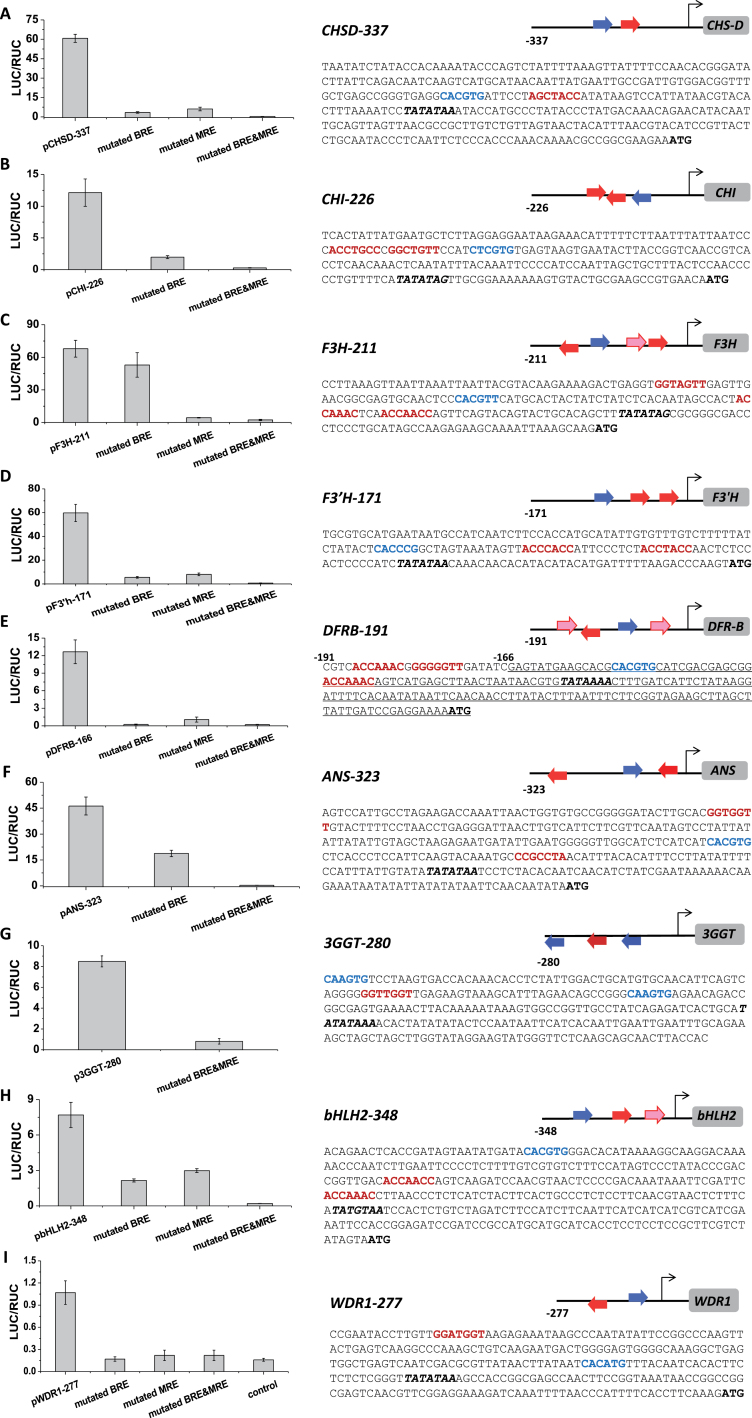
Proximal promoter architectures of the *I. purpurea* anthocyanin pathway genes. (A–I) Test results of the 5′-noncoding regions of the anthocyanin genes from *IpCHS-D* to *IpWDR1*. Each gene is displayed by its dual luciferase results on the left and features of the proximal promoter sequence on the right (see Fig. 3 and Supplementary Figure S3 for mutation details). The proximal regions are marked at the sites of experimentally confirmed BREs (in blue and bold) and MREs (in red and bold, along with the expected TATA box (in italic and bold)). The mutated MREs and BREs shown on the *x*-axis on the left correspond to those confirmed *cis* elements on the right. The orientations of the *cis* elements are cartooned by the insert figure with arrows representing the *cis* motifs for each gene. The arrows are coloured in accordance with those of MREs and BREs; and those above the line and pointing to the translation start site are oriented following the syntax and the ones below the line are in the complementary layout. The pink arrows are for the weak MREs. The angled arrow shows the start site for the transcription of the gene. The box indicates the coding region. The numbers above the sequences show the relative positions from the translation start site, and those after the gene names the lengths of the regions.

Collectively, transient and EMSA results led to identification of the major *cis* elements over the anthocyanin pathway (Fig. 4), including combined identification of MRE and BRE on the *Ip3GGT* promoter. These valid motifs indicated that the orientation of the *cis* elements could change, as could the numbers of MREs and BREs. The presence of both MRE and BRE is essential for the promoters to respond to IpMBW. In addition, variants of some of the *cis* elements identified were also functional, as tested on *IpCHSD-337*_*pro*_ (Supplementary Figure S5). Putting all the information on *cis* motifs together, we found they converged on ANCNNCC for MREs and CACN(A/T/C)(G/T) for BREs (Table 1). The two syntaxes representing nine anthocyanin pathway genes indicate a *cis* logic shared by the pathway. As the two syntaxes all fall in the proximal promoter regions typically within 350bp from the translation start site (Supplementary Figure S1C), the pathway genes appear to have a tight motif organization in the proximal region of a promoter in common.

**Table 1.** Experimentally confirmed *cis* elements on anthocyanin gene promoters

Method	Species	Gene	BRE	BRE location	MRE	MRE location
Luciferase assay and EMSA	*I. purpurea*	*CHS-D*	CACGTG	205–200	AGCTACC	193–187
					ACCCACC	321–315
		*CHI*	CACGAG	143–148	ACCTGCC	167–161
					*AACAGCC*	153–159
		*F3H*	CACGTT	135–130	*AACTACC*	160–166
					ACCAAAC (weak)	97–91
					ACCAACC	87–81
		*F3′H*	CACCCG*	106–101	ACCCACC	86–80
					ACCTACC	70–64
		*DFR-B*	CACGTG	152–147	ACCAAAC (weak)	187–181
					*ACCCCCC*, *ATCAACC*	170–180
					ACCAAAC (weak)	133–127
		*ANS*	CACGTG	155–150	*AACCACC*	265–271
					*ATCCGCC*	117–123
		*3GGT*	*CACTTG*	275–280, 177–182	*ACCAACC*	212–218
		*bHLH2*	CACGTG	321–316	ACCAACC	226–220
		*WDR1*			*ACCATCC*	257–263
EMSA	*I. purpurea*	*3GT*			ACCTAAC	105–99
	*Z. mays*	*Bz (3GT*)			ACCTGCC	Wang *et al.*, 2013
		*A1 (DFR*)			ACCTACC	
	*Malus domestica*	*MYB10*			*AGCTACC*	
	*Gerbera hybrida*	*DFR2*			AACTTAC	
Luciferase assay	*I. purpurea*	*CHSD-337*_*pro*_	cacgtt		agcaacc	mutation
			cacgcg		agcgacc	mutation
Consensus			**CACN(A/C/T)(G/T**)		**ANCNNCC** (ANCNNAC weak)	

The numbers for location are relative to the translation start site of the gene. Sequences underlined are ones in the reverse or/and complementary form on the promoters. Those in lower case were tested on mutated *IpCHSD-337*_*pro*_. *, Little binding to IpbHLH2 in EMSA.

### Generality and layout pattern of *cis* elements on the anthocyanin gene promoters

**Fig. 5.**
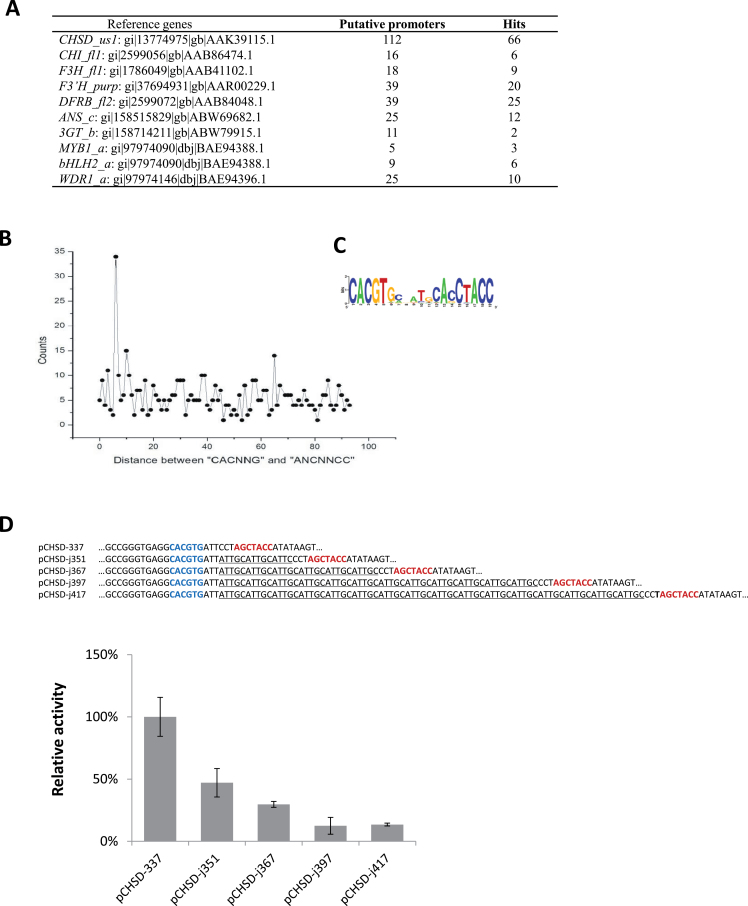
A common layout for the BRE and MRE on the anthocyanin gene promoters across species. (A) Results of a bioinformatic survey. The column for putative promoters lists the numbers of 5′-noncoding sequences with their coding regions sharing >40% amino acid identity with those of *I. purpurea* anthocyanin genes. Of these sequences, the number of promoter sequences having the specific arrangement of BREs and MREs [from 5′ to 3′ as CACNNG(N_1–100_)ANCNNCC] is listed as hits for each gene. (B) Frequency distribution of the distance (bp) between BRE and MRE candidates detected on the 159 promoter sequences. The *cis* candidates were within 100bp of each other (Supplementary Table S4). The sequences are 200–2000bp long, covering the 5′-noncoding region upstream of the translation start site. (C) The *cis* motifs reported by MEME for the peak in (B). The peak consists of 34 sequences. (D) Effect of between-*cis* distance on promoter activity. The distance alterations were carried out on *IpCHSD-337*_*pro*_. All five constructs had the same *cis* elements (in bold, MRE to the right and BRE to the left) but different spacer lengths to separate the distances between the *cis* elements. The effect of changing the distance was analysed in the transient expression system driven by the IpMBW complex. The activity of the native promoter estimated in LUC/RUC was set as 100%. The error bars are from three replicates. This figure is available in colour at *JXB* online.

We searched for the syntaxes in multiple species to test for their generality. Searches using the blastp command led to the identification of available coding sequences of anthocyanin genes in the NCBI database after taking those of *Ipomoea* genes as templates (Fig. 5A). A collection of 299 anthocyanin gene promoter sequences was retrieved. A fixed *cis* layout pattern (CACNNG (N_1–100_)ANCNNCC) was taken as the search criterion for these sequences, as the pathway-based identification of *cis* motifs suggested a common layout of BRE and MRE (5′→3′) on the anthocyanin genes (Fig. 4). From the collected promoter sequences, 159 promoters of 35 species (53%) were found exhibiting the pair of *cis* elements within 100bp of each other. The species included *Ginkgo biloba*, monocots and dicots (Supplementary Table S4). Interestingly, the distance between the *cis* elements showed a peak at 6-bp apart (Fig. 5B). The sequences giving rise to the peak showed two significant motifs agreeing with the syntaxes identified in *Ipomoea* and *Arabidopsis* (Fig. 5C). As *IpCHSD*_*pro*_ hosts the BRE and MRE at this preferred distance of 6-bp apart (Fig. 4A), the biological significance of the distance can be evaluated in transient expression assays. By artificially increasing the between-*cis* distance, we found that the distance affected the promoter activity negatively. Lengthening the distance (up to 60bp) significantly weakened the promoter strength; nevertheless, about 12% of the promoter activity still remained even after the distance was increased further to 80-bp apart (Fig. 5D).

### The regulatory specificities of MBW complexes

**Fig. 6.**
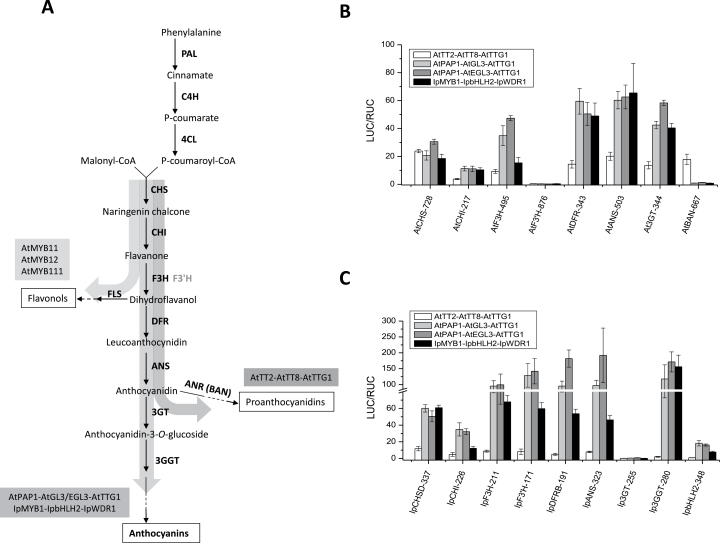
Comparisons of the flavonoid network regulation between *Arabidopsis* and *Ipomoea*. (A) The anthocyanin pathway backbone and the major branches. The arrows and associated TFs indicate the known regulated segments of the network after this work and that of Stracke *et al.* (2007). Pathway regulation by four MBW complexes on the flavonoid genes of *A. thaliana.* (B) and *I. purpurea* (C) were measured in three replicates in dual luciferase assays. The 5′-noncoding regions of the genes are labelled along the *x*-axis. The normalized promoter activities (in LUC/RUC) are indicated by the *y*-axis.

Since both *trans* and *cis* components are vital for the regulatory module formed at the 5′-noncoding region, we evaluated the specificities of the *trans* factors in governing the transcription of flavonoid genes. This analysis included proximal promoters of ten flavonoid genes (*CHS*, *CHI*, *F3H*, *F3′H*, *DFR*, *ANS*, *3GT*, *AtBAN, Ip3GGT*, and *IpbHLH2*) and four sets of MBW complex from two species (*Arabidopsis* and *I. purpurea*). Three TF complexes [AtTT2-AtTT8-AtTTG1 (AtTTT), AtPAP1-AtGL3-AtTTG1 (AtPGT), and AtPAP1-AtEGL3-AtTTG1 (AtPET)] were from *Arabidopsis*, and one (IpMBW) from *I. purpurea*. We found that AtPGT, AtPET, and IpMBW could positively regulate all tested genes except *AtF3′H*, *AtBAN* and *Ip3GT*. In comparison, AtTTT also activated the same set of genes (except *IpbHLH2*), in addition to *AtBAN* (Fig. 6), congruent with its role in PA synthesis; the activation intensity was lower than AtPGT and AtPET for the same constructs. While all TF complexes were capable of regulating *CHS*, *CHI*, and *F3H*, none could activate *AtF3′H* or *Ip3GT*. A close search of the 876-bp long 5′-noncoding region of *AtF3′H* indicated that MREs and BREs were both absent within the region about 500-bp upstream of the translation start site (Supplementary Figure S6), despite typical BRE and MRE sequences being located at –659 and –584 from the translation start site, respectively. Both *Ip3GT* and *IpMYB1* were found lacking BREs in their proximal promoters (Supplementary Figure S6), which explained their low promoter activities in the presence of IpMBW (Fig. 1B). A common pattern in the TF complexes such as IpMBW, AtPGT, and AtPET was that they all demonstrated strong power of activation towards the later enzyme genes of the anthocyanin pathway (*DFR*, *ANS*, and *At3GT*). In contrast, AtTTT could activate these genes at a relatively low level (Fig. 6).

After excluding *AtF3′H* and the more-or-less redundant AtPET (relative to AtPGT), we analysed the transient expression data in two-way ANOVAs. The analysis suggests that promoter identity is the leading factor for proximal promoter activity on the anthocyanin pathway, and the interactions between the TF complex and promoter (TF × promoter) plays a role as significant as the TF complex itself (Table 2). When the analysis was performed again with the transient expression data of only *Arabidopsis* or *Ipomoea*, the pattern remained the same. Proximal promoters are hence considered the major force governing gene transcription in the flavonoid network, irrespective of species.

**Table 2.** Analysis by two-way ANOVA on pathway promoter activities of flavonoid genes

Source	df	Mean square	*F*-value	Pr > *F*	Power
Promoter	7	1.58	101.19	< 0.0001	0.999
TF complex	2	0.43	27.20	< 0.0001	0.999
Promoter × TF complex	14	0.46	29.47	< 0.0001	0.999

Eight proximal promoters included *AtBAN*, *At3GT*, *AtANS*, *AtDFR*, *AtF3H*, *AtCHI*, *AtCHS*, and *IpCHS-D*. Three TF complexes were from *Arabidopsis* (TT2-TT8-TTG1, PAP1-GL3-TTG1) and *I. purpurea* (IpMYB1-bHLH2-WDR1). Each promoter activity (log transformed) was measured by three replicates of its transient expression (*R* ^2^ = 0.96, *n* = 72). The *F*-value was from *F*-test on the effect of each variable (source).

## Discussion

Our analysis has provided a bilateral view of pathway regulation, with the molecular mechanism discussed in both promoter architecture and TF–*cis* interactions featuring the MBW complex. The promoter architecture was found following a common logic for the anthocyanin pathway, including the orientation of the BRE and MRE within the proximal promoter and the semi-conservative composition of *cis* elements for MYB and bHLH. The syntaxes for both BRE and MRE contain highly variable nucleotide sites, which may lead to segmented summaries for the binding sites of the complex. For instance, for 85 R2R3 MYBs of the *Arabidopsis* genome, three categories of MYB binding site (MBS) were suggested (Romero *et al.*, 1998). MBSI (CNGTTR, or T/CAACNG) partially overlaps with MBSII [GTTWGTTR, or T/CAAC(T/A)A(C/A)C], while MBSIIG [GKTWGGTR, or C/TACC(T/A)A(C/A)C] differs from MBSII by one nucleotide. The latter two also agree largely with the AC-rich element [A/CCC(T/A)A(C/A)C/G] proposed by Hartmann *et al.* (2005). Our summary of MREs for MYB1- like homologues in the syntax of ANCNN(C/A)C appears to unify these categories in both length and content, providing a basis for future expanded inquiries on R2R3 MYBs in the plant genome. In this study, however, we mainly focused on the anthocyanin pathway, showing that a systematic approach could enable a deeper understanding of the regulatory mechanism in the context of the related flavonoid branches.

### MBW regulates the pathway genes as one unit

Identification of *cis* elements in *I. purpurea* pathways led to a mechanistic understanding of how TFs regulate groups of genes. In both *Arabidopsis* and *Ipomoea,* transcription of the enzyme-coding genes of the anthocyanin pathway were activated as one unit, just like the pathway regulation found in maize (Carey *et al.*, 2004). Since AtPGT and AtPET activate the same set of genes as IpMBW does, their orthology can be established in functioning as *bona fide* regulators of the anthocyanin pathway. Interestingly, the PA pathway in *Arabidopsis* appears to be regulated together with the anthocyanin pathway, as shown by the wide-ranging regulation capacity of AtTTT on all the anthocyanin pathway genes (with the exception of *AtF3′H* but including *At3GT*) and *AtBAN*. The result agrees with reported regulation of AtTTT on *AtCHS* (Appelhagen *et al.*, 2011), *AtDFR*, *AtANS*, *AtBAN* (Baudry *et al.*, 2004; Xu *et al.*, 2014a), and additionally *AtCHI* and *AtF3H* (Xu *et al.*, 2014b). Against this background, all tested MBWs failed to activate *AtF3′H* and *Ip3GT* in the transient expressions, consistent with their lack of the appropriate *cis* elements in the proximal promoters. These genes are either regulated independently of MBW or need extra regulatory components beyond the proximal regions. For instance, the grape *UFGT* was considered to be regulated independently of the other genes (Boss *et al.*, 1996), and *ZmA1* required the presence of R-interacting factor 1 to be activated by its conspecific MBW (Kong *et al.*, 2012). Regardless, the cases of *AtF3′H* and *Ip3GT* suggest that both an MRE and a BRE are required for the MBW complex to activate the promoter of a target gene after the local chromatin structure is cleared. Most pathway genes identified so far do contain the *cis* regulatory region with the necessary footings for the MBW complex.

### Parallel redundancy of *cis* elements and multiple TFs feature combinatory regulation

The pathway genes show multiple copies of each type of *cis* element (particularly those of MREs). At least three functional MREs were experimentally confirmed on each of *IpCHI-226*_*pro*_, *IpF3H-211*_*pro*_ *, IpF3′H-171*_*pro*_, and *IpDFRB-191*_*pro*_ (Fig. 3; Fig. 4; Supplementary Figure S3). For instance, if one of the MREs was mutated on *IpDFRB-191*_*pro*_, no phenotypic change could be found on the seedlings transformed with the mutated construct. Although refractory in experimental validations of the *cis* elements involved, the redundancy in binding sites may provide dynamics as well as intricacy to the gene transcription. A full occupancy of the *cis* sites in a promoter region, for instance, may result in a higher level of gene transcription than is the case for partial occupancy of the *cis* sites.

The multiple *cis* elements also mirror functional redundancy of TFs. Noticeably, AtTTT activates the same set of anthocyanin pathway genes as AtPGT and AtPET do, but possibly at a lower capacity (Fig. 6). This may explain why the transgenic *Arabidopsis* plants showed accumulation of anthocyanins but no obvious PA accumulation in the cases of the short promoter sequences (Fig. 2C). The level of PA synthesis in these cases was probably too low to be visible. Given the restricted yet high expression of *AtTT2* in developing seeds (Nesi *et al.*, 2001) and a low expression of *AtPAP1* in developing siliques (Matsui *et al.*, 2004), the extensive control from *AtCHS* to *AtBAN* by the AtTTT complex on the flavonoid network allows independent PA synthesis in the seed coat. Likewise, flavonol synthesis appears to use the same principle, where *AtCHS*, *AtCHI*, and *AtF3H* are regulated by AtMYB11, AtMYB12, and AtMYB111, which also target the flavonol synthase 1 gene (Stracke *et al.*, 2007). The regulatory redundancy of the same genes may offer insurance to different functional branches of the network. For TF redundancy, maize *B* alleles can substitute for *R* in inducing the expression of *Zm3GT* (Goff *et al.*, 1990). Also, the triple mutant of *AtMYB11*, *AtMYB12*, and *AtMYB111* still accumulates anthocyanin (Stracke *et al.*, 2007), but *AtPAP1*has little to do with PA accumulation (Gonzalez *et al.*, 2008). The redundancy in both *cis* and *trans* factors in pathways enables a cell-specific regulation of the same pathway by different arrays of regulatory proteins.

### Promoter architecture is the most important factor for pathway regulation

Promoter architecture is believed to take a central position in transcription (Spitz and Furlong, 2012), but detailed studies have been scarce. A promoter’s architecture is characterized here by the distribution of all *cis* elements in the region, including the binding sites of TFs and the basal transcription apparatus (such as the TATA box), numbers of *cis* elements, spatial distance between *cis* elements, and the relative orientations of *cis* elements. These factors may significantly affect a promoter’s activity. For the anthocyanin pathway genes, the *cis* regulatory sites summarized by the syntax identified for MREs (ANCNNCC) overlap with several previously postulated motifs. For example, cctacc(n)7ct, described as an H-box (Loake *et al.*, 1992), shows an overlap of NCNNCC, and the consensus (AC)ACC(TA)A(AC)C described by Sablowski *et al.* (1994) is also mostly congruent with the MRE syntax here; in the case of TTGACTGGnGGnTGCG for C1 binding (Lesnick and Chandler, 1998), GGnGGnT is clearly the reverse complement of ANCNNCC. The syntax also includes ANCNACC, shown in a recent consensus summary (Wang *et al.*, 2013). We have shown here that both orientations of the syntaxes are valid on the anthocyanin genes (Table 1). The intermittently conserved sites within the MRE syntax have greatly increased the difficulty of *cis* element predictions. Even when all the existing data support the syntax for MREs, for instance, it provides still more of guidance than a total replacement of experimental validations since a fraction of cases that obey the structure of the syntax have been proven nonfunctional, at least to IpMBW (Supplementary Figure S5). The same is also true for the syntax of BREs. It matches well with the previous G-box (Staiger *et al.*, 1991; Loake *et al.*, 1992) but not the E-box (CANNTG). Certain cases satisfying the syntax such as CACTTG were proven invalid for IpbHLH2 (Supplementary Figure S2). The mutation tests of Bodeau and Walbot (1996) suggested that the R-motif in the form of CACGAC and CACGAG was effective on the *Bz2* (GST) promoter, suggesting that the BRE syntax holds for both monocots and dicots.

The proximity of BRE and MRE in the 5′-noncoding region was revealed by both the promoter survey (Fig. 5) and identification of the *cis* motifs over the anthocyanin pathway (Fig. 4). The influence of *cis* spacing on promoter activity was previously indicated in the *rbcS* promoter (Lopez-Ochoa *et al.*, 2007), and further analysed here to show its relevance to transcriptional intensity. Locations of most MREs on the anthocyanin genes were found close to the translation start site; BREs, on the other hand, are largely located 5′ to the first MRE (Fig. 4). This order of BRE and MRE from 5′ to 3′ in the noncoding region is not only broadly found on the anthocyanin gene promoters (> 53%) but also present in the promoter of *Rd22*, an abscisic acid-mediated dehydration-responsive gene (Abe *et al.*, 2003). The occurrence of the *cis* orientation in different molecular systems driven by MYB and bHLH implies an antiquity of the *cis* arrangement for genes regulated by bHLH and MYB.

We have shown that promoter architecture varied greatly among genes, facilitating variations of gene expression across pathways that is regulated by the same set of TFs. The regulatory mechanism of the anthocyanin pathway is mostly about the cooperation of the *cis*-regulatory elements and the cognitive TF complex, taking the same logic of TF–DNA interactions across the pathway. The findings have led to greater understanding of the regulatory mechanism of the anthocyanin pathway and the opportunity for more quantitative analysis of TF–DNA interactions. It will be interesting to see how other TFs affect pathway gene regulation.

## Supplementary material

Supplementary data can be found at *JXB* online.

Supplementary Table S1. List of primers used in the experiments in this study.

Supplementary Table S2. Accessions of genes and promoter sequences.

Supplementary Table S3. Correlation coefficients of transcript levels by RT-qPCRs.

Supplementary Table S4. List of 159 sequences of the anthocyanin genes at the 5′-noncoding region.

Supplementary Figure S1. Characterization of *Ipomoea* TFs and promoter activities of the target genes.

Supplementary Figure S2. Binding capacity of IpbHLH2 in EMSAs.

Supplementary Figure S3. Analysis of *cis* elements on the anthocyanin proximal promoters of *I. purpurea*.

Supplementary Figure S4. Comparisons of TF dosages in dual luciferase assays.

Supplementary Figure S5. Mutations on *CHSD-337*_*pro*_ showing DNA binding spectrums of IpMYB1 and IpbHLH2.

Supplementary Figure S6. 5′-noncoding sequences of flavonoid genes of two species (*A. thaliana* and *I*. *purpurea*).

Supplementary File S1. Bioinformatic survey with Perl scripts and instructions.

## Funding

The study was supported by National Science Foundation of China (91331116, 31070263) and Chinese Academy of Sciences (KSCX2-YW-N-043).

## Supplementary Material

Supplementary Data
