# Supplementary material for: Characterization of the cis elements in the proximal promoter regions of the anthocyanin pathway genes reveals a common regulatory logic that governs pathway regulation
Source: J Exp Bot. 2015 Apr 23;66(13):3775–89. doi: 10.1093/jxb/erv173 (PMC4473980; doi:10.1093/jxb/erv173)
Supplement: Supplementary Data [file supp_66_13_3775__index.html]

Characterization of the cis elements in the proximal promoter regions of the anthocyanin pathway genes reveals a common regulatory logic that governs pathway regulation — Characterization of the cis elements in the proximal promoter regions of the anthocyanin pathway genes reveals a common regulatory logic that governs pathway regulation — Supplementary Data 

# Characterization of the *cis* elements in the proximal promoter regions of the anthocyanin pathway genes reveals a common regulatory logic that governs pathway regulation

## Supplementary Data

Data files

**Files in this Data Supplement:**

- Supplementary Data - Supplementary Data
