# Supplementary material for: Characterization of the cis elements in the proximal promoter regions of the anthocyanin pathway genes reveals a common regulatory logic that governs pathway regulation
Source: J Exp Bot. 2015 Apr 23;66(13):3775–89. doi: 10.1093/jxb/erv173 (PMC4473980; doi:10.1093/jxb/erv173)
Supplement: Supplementary Data [file supp_erv173_jexbot145573_file001.pdf]

Supplementary files to the following article

4 tables and 6 figures

Pathway - wise characterization of the *cis* elements in the proximal promoter regions of the anthocyanin pathway genes reveals a common regulatory logic that governs pathway regulation

Zhixin Zhu[1,2], Hailong Wang [1,2], Yiting Wang[1,2], Shan Guan[1], Fang Wang[1,2], Jingyu Tang[1,2], Lulu Xie[1,2], Yingqing Lu[1,2,*]

[1] State Key Laboratory of Systematic and Evolutionary Botany, Institute of Botany, Chinese Academy of Sciences, 20 Nan Xin Cun, Beijing 100093, China
[2] University of Chinese Academy of Sciences, Beijing 100049, China

Supplementary Table S1. List of primers.

Experiment	Gene	Primer name	Sequense (5’-3’)
EMSA	*IpMYB1*	Ipmyb1-BamHI-F	TATGGATCCATGGTTAATTCTTCTGCAAGGTG
		Ipmyb1-XbaI-R	TATTCTAGATTAAATGGTTGTGTCTAAAAG
	MlMYB1	Mlmyb1-BamHI-F	AATGGATCCATGCATGAAAAGTCGCAGTTGGGG
		Mlmyb1-SalI-R	ATAGTCGACTTAAGTGTCAACCAAACAACGGCCTAC
	IpbHLH2$^{462-528}$	bHLH2-a1383-BamHI-F	TTGGATCCGAAGAGCCCAACGTCAACCAC
		bHLH2-a1584-HindIII-R	ATAAGCTTCTGCCGGTCAACCTCCGTC
Yeast two hybrid	*IpMYB1*	IpMyb1-XholR-A	TATCTCGAGTTAAATGGTTGTGTCTAAAAG
		IpMyb1-BamHIF-A	TATGGATCCATGGTTAATTCTTCTGCAAG
		IpMyb1-XhoF+B	TATCTCGAGCAATGGTTAATTCTTCTGCAAG
		IpMyb1-PstR-B	TATCTGCAGTTAAATGGTTGTGTCTAAAAG
	IpbHLH2	IpBH2-XholR-A	GGACTCGAGCTAAAACTGAGGAATTATRCTATG
		IpBH2-BamHIF-A	GATGGATCCATGGCGGAAACCCCTG
		IpBH2-SalF+B	GATGTCGACTAATGGCGGAAACCCCTG
		IpBH2-SalR-B	GGAGTCGACCTAAAACTGAGGAATTATRCTATG
	IpWDR1	lpWD1-XholR-A	GATCTCGAGTTACACTTTTAGCATCTGAAGCTTG
		lpWD1-BamHIF-A	GATGGATCCATGGTGAACTCAACCCAAGG
		lpWD1-SalF+B	GATGTCGACTAATGGTGAACTCAACCCAAGG
		lpWD1-SalR-B	GATGTCGACTTACACTTTTAGCATCTGAAGC
Subcellular localization	*IpMYB1*	IpMYB1HindIII-F	GACAAGCTTATGGTTAATTCTTCTGCAAGG
		IpMYB1BamHI-R	GACGGATCCTTAAATGGTTGTGTCTAAAAG
		M-cfp-Hind3F	TATAAGCTTATGGTGAGCAAGGGCGAGGAG
		M-cfp- Hind3R	TATAAGCTTCTTGTACAGCTCGTCCATGCCG
	IpbHLH2	IpbHLH2SalI-F	TATGTCGACGATGGCGGAAACCCCTGGCG
		IpbHLH2BamHI-R	GCCGGATCCCTAAAACTGAGGAATTATRCTATG
		B-yfp-SalI-jia-F	TATGTCGACGATGGTGAGCAAGGGCGAGGAG
		B-yfp-SalI-jia-R	TACGTCGACCTCTTGTACAGCTCGTCCATGCCG
	IpWDR1	IpWDR1SalI-F	TATGTCGACGATGGTGAACT CAACCCAAGG
		IpWDR1BamHI-R	GCCGGATCCTTACACTTTTAGCATCTGAAGC
		mRFP Hind3-F	GGAAGCTTATGGCCTCCTCCGAGGACGTCATC
		mRFP SalI-R	TAGTCGACGGCGCCGGTGGAGTGGC
QPCR	*IpMYB1*	MYB1-540	GCTAAGGCTGTCGTCTATG
		MYB1-810	GGTCCACATCAATCGGAAAG
	IpbHLH2	BH2-970	GTATTCGGGTGGGAACG
		BH2-1270	TGGAACAGGTGGAGCTG
	IpWDR1	WD-290	GTCAATGAAGTCCGCCG
		WD-525	TCAATTGGGTTTCCACGACG
	IpCHS-D	CHSD-D2FOR	TGGGACGCTATGGAGGAC
		CHSD-D3P	GAACAACATCGAGAAATGCTTGTCGG
	IpCHI	CHI-450	CGCCTCTATCTTCTTCACTC
		CHI-710	CGACCGTTTGTGGAATGAC

	IpF3H	F3H-370	GGTGAAGCAGTGAAAGATTGG
		F3H-580	GTCCATTTCCACACAGGCTT
	IpF3′H	F3′H-600	GACCCAAAGGCGGAGGAG
		F3′H-880	TCCCTCTTCGCTATCCGTATCAG
	IpDFR-B	DFR-760	GCAGAAGGAAGATTCATCTGCT
		DFR-1030	GCTCTTCTTCAATTGCAGCAG
	IpANS	ANS-850	GCAACGGCAAGTACAAGAG
		ANS-1110	GATTGATGATCATCATCATTATCAGG
	Ip3GT	3GT-1114	CCATTCTATGGGGATCAGCA
		3GT-1356	AAGCAGGTGCACTAATTCTTGGAA
Transient expression assays for *Ipomoea purpurea*	*LUC*	LUCNcoI-F	TATCCATGGAAGACGCCAAAAACATAAAG
		LUCEcoRI-R	TATGAATTCTTACACGGCGATCTTTCCGCCC
	RUC	RUCHindIII-F	GACAAGCTTATGACTTCGAAAGTTTATGATC
		RUCEcoRI-R	GCCGAATTCTTATTGTTCATTTTTGAGAAC
	IpMYB1	IpMYB1HindIII-F	GACAAGCTTATGGTTAATTCTTCTGCAAGG
		IpMYB1BamHI-R	GACGGATCCTTAAATGGTTGTGTCTAAAAG
		myb-KpnI-375F	TAGGTACCGAGGAGACTCGTGGCAGAC
		myb-trans-BamHI-R	TAGGATCCGGCAGGCTGACGTAAATTGCTTA
	IpbHLH2	IpbHLH2SalI-F	TATGTCGACGATGGCGGAAACCCCTGGCG
		IpbHLH2BamHI-R	GCCGGATCCCTAAAACTGAGGAATTATRCTATG
		bh2a-KpnI-348F	ACAGGTACCACAGAACTCACCGATAGT
		bh2a-KpnI-976F	AGAGGTACCCATTAAGCCACCTTTTCTGA
		bH2-trans-BamHI-R	TGCGGATCCTACTATAGACGAAGCGGAGG
	IpWDR1	IpWDR1SalI-F	TATGTCGACGATGGTGAACT CAACCCAAGG
		IpWDR1BamHI-R	GCCGGATCCTTACACTTTTAGCATCTGAAGC
		WD-KpnI-277F	AGAGGTACCGAATACCTTGTTGGATGG
		WD-KpnI-1554F	AGAGGTACCAACCCGCAGTCACTATCA
		WD-trans-BamHI-R	TGCGGATCCTTTGAAGGTGAAAATGGGTTA
	IpCHS-D	chs-d-KpnI-337F	TGAGGTACCCTAATATCTATACCACAAAATACCC
		chs-d-KpnI-1067F	TGAGGTACCTTCATACCATCCATACACCGT
		chs-d-NcoI-R	ACACCATGGTTCTTCGCCGGCGTTTTGTTTG
	IpCHI	CHI-KpnI-226F	GACGGTACCTCACTATTATGAATGCTCTT
		CHI-KpnI-751F	AGAGGTACCGCATAGGTGGGTTCACAAC
		CHI-SalI-R	TGAGTCGACTGTTCACGGCTTCGCAGTAC
	IpF3H	F3h-KpnI-211F	TGAGGTACCTTAAAGTTAATTAAATTA
		F3H-KpnI-461F	GACGGTACCAACAACTCACACAACAACTC
		F3H-KpnI-1780F	AGAGGTACCGACAATAATCATTTCATAAGGT
		F3H-sal-R	TGAGTCGACTTGCTTTAATTTTGCTTCTCTTGG
	IpF3′H	F3'h-KpnI-171F	TGAGGTACCTGCGTGCATGAATAATGCCA
		F3'h-KpnI-663F	TGAGGTACCTTGACATTTTTTACCAGAAGTT
		F3'h-SalI-R	TGAGTCGACACTTGGGTCTTAAAAATCATGT
	IpDFR-B	dfr-KpnI-136F	TGAGGTACCGGACCAAACAGTCATGAGC
		dfr-KpnI-166F	TGAGGTACCGAGTATGAAGCACGCACGT

		dfr-KpnI-191F	TGAGGTACCGTCACCAAACGGGGGGTTGAT
		dfr-KpnI-236F	TGAGGTACCATTATTGTTGGAATAATATAT
		dfr-KpnI-314F	TGAGGTACCTTAAGCATTGAAGTGTCG
		dfr-KpnI-1559F	TGAGGTACCCACACCAATTGCAAATCTA
		dfr-NcoIr	CTGCCATGGTTTTCCTCGGATCAATAAGCT
	IpANS	ans-kpnI-323F	AGAGGTACCAGTCCATTGCCTAGAAGACCAAAT
		ans-BamHI-R	TGCGGATCCTATATTGTTGAATTATATATAATA
	Ip3GT	U-kpnI-255F	GACGGTACCTTCATAGTAACCAAAAGAGA
		U-kpnI-1007F	ATAGGTACCCGCCAGCGCCCAAATGCAA
		Uc-kpnI-235F	TAGGTACCAATTTCAATTTTGGTCACACGA
		Uc-kpnI-480F	TAGGTACCGCTTATTCCCTGTCATCAA
		Uc-kpnI-732F	TAGGTACCGCAAGCCAAGAAGACTTCAATA
		U-kpnI-930F	ATAGGTACCTTTCCCGTCCCAATGAAT
		U-sal-R	GAGGTCGACTTTTTCCGGCGAGAAATGGTT
	Ip3GGT	3ggt-KpnI-280F	ACCGGTACCCAAGTGTCCTAAGTGAC
		3ggt-BamHI-R	TTGGATCCGTGGTAAGTTGCTGCTTGAG
Transgenic primers for *Arabidopsis tt3* transformations	*IpDFR*	gDFR-KpnIF	CGAGGTACCATGGTGGACGGTAATCATCCT
		gDFR-SacIR	CTGAGCTCTCAAGCTTTTAAGGGCACTAC
		proDFR1559-PstIF	GATCTGCAGCTTTGCCACATAACCCACCAT
		pDFR191-PstIF	TTCTGCAGCGTCACCAAACGGGG
		pDFR166-PstIF	TTCTGCAGGAGTATGAAGCACG
		proDFR-KpnIR	CTAGGTACCTTTTCCTCGGATCAATAAGCT
Transient expression assays for *Arabidopsis thaliana*	*AtTT2*	TT2Hind3F	CCAAGCTTATGGGAAAGAGAGCAACTACTAGTGTGAGGAGAGAA
		TT2SalIR	AAGTCGACTCAACAAGTGAAGTCTCGGAGCCAATCTT
	AtTT8	TT8Hind3F	CCAAGCTTATGGATGAATCAAGTATTATTCCGGCAGAGAAA
		TT8SalIR	CAGTCGACCTATAGATTAGTATCATGTATTATGACTTGGTGGAT
	AtTTG1	TTG1SalIF-jia	ACGTCGACAATGGATAATTCAGCTCCAGATTCGTTATCCAGA
		TTG1EcoRI-R	CCGAATTCTCAAACTCTAAGGAGCTGCATTTTGTTAGCA
	AtPAP1	PAP1Hind3F	AGAAGCTTATGGAGGGTTCGTCCAAAGGGCTGCGAAAAGGTG
		PAP1SalIR	CCGTCGACCTAATCAAATTTCACAGTCTCTCCATCGAAAAGA
	AtGL3	GL3SalIF-jia	CCGTCGACCATGGCTACCGGACAAAACAGAACAACTGTGCCAGAGA
		GL3EcoRI-R	CCGAATTCTCAACAGATCCATGCAACCCTTTGAAG
	AtEGL3	EGL3SalIF-jia	CCGTCGACCATGGCAACCGGAGAAAACAGAACGGTGCCGGACAAT
		EGL3EcoRI-R	AAGAATTCTTAACATATCCATGCAACCCTTTGAAGTGCCTCTT
	AtCHS	AtCHS-728KpnIF	ACGGTACCGTTGGAAAGCGCAAATAGGG
		AtCHS-SalIR	CAGTCGACTATAGTATACACCAACTTGGGTTTA
	AtCHI	AtCHI-217KpnIF	ACGGTACCGCTCAAAGTTTCAACCACCAA
		AtCHI-BamHIR	ACGGATCCTGTTGAGTCGGTTGGAATTTCG
	AtF3H	AtF3h-495KpnIF	ACGGTACCGCAAGCCCGTACCAGAACAT
		AtF3h-SalIR	ACGTCGACTGTAATTACGAAGACAAAAGACTA
	AtF3'H	AtF3pH-876KpnIF	AAGGTACCAAACTAATGAACTGTAACCTCTT
		AtF3pH-SalIR	AAGTCGACAGTGTTGGGTTTGAATGGTAAGT
	AtDFR	AtDFR-343KpnIF	AAGGTACCTGAACTGAAGTCACCCACACGTCT

	AtDFR-SalIR	CAGTCGACTTTTGTGGTTATATGATAGAT
AtANS	AtANS-503KpnIF	ACGGTACCTCCAAACTACAAAAAGAAAATG
	AtANS-SalIR	CCGTCGACCTTCTTTAGTCTTCTGTTTAAAGCTA
At3GT	At3GT-344KpnIF	AAGGTACCAGCGTAACGAACAAATAACGAC
	At3GT-BamHIR	CAGGATCCTTTCTTGGACTCTCTTGTATTTC
AtBAN	AtBAN-667KpnIF	AAGGTACCGAATGCTATTGCCAATGCCTTC
	AtBAN-SalIR	AAGTCGACGATTGTACTTTTGAAATTACAGAGAT

The DNA restrcition enzyme cutting sites are underlined.

Supplementary Table S2. Accessions of genes and promoter sequences investigated in this study.

Species	Gene	Seq ID	Promoter seq ID
Ipomoea purpurea	*CHS-D*	AF358654	AF358654
	CHI	EU032606	KC794944
	F3H	U74081	KC794947
	F3'H	AY333419	AB113265
	DFR-B	AB018438	KP053926
	ANS	EU032614	KC794942
	3GT	AF028237	KC794941
	3GGT	KC794956	KC794952
	MYB1	AB232769	KC794943
	bHLH2	EU032618	KC794946
	WDR1	AB232777	KC794945
Arabidopsis thaliana	*CHS*	AT5G13930	AT5G13930
	CHI	AT3G55120	AT3G55120
	F3H	AT3G51240	AT3G51240
	F3'H	AT5G07990	AT5G07990
	DFR	AT5G42800	AT5G42800
	ANS(LDOX)	AT4G22880	AT4G22880
	UF3GT	AT5G54060	AT5G54060
	BAN	AT1G61720	AT1G61720
	PAP1	AT1G56650	AT1G56650
	TT2	AT5G35550	AT5G35550
	GL3	AT5G41315	AT5G41315
	EGL3	AT1G63650	AT1G63650
	TT8	AT4G09820	AT4G09820
	TTG1	AT5G24520	AT5G24520

Supplementary Table S3. Pearson correlation coefficients among transcript levels of anthocyanin pathway genes.

	IpCHS-D	*IpCHI*	*IpF3H*	*IpF3'H*	*IpDFR-B*	*IpANS*	*Ip3GT*	*IpMYB1*	*IpbHLH2*	*IpWDR1*
IpCHS-D		**0.856**	**0.921**	**0.561**	**0.840**	**0.887**	**0.643**	**0.903**	**0.840**	**0.762**
IpCHI	0.001		**0.906**	**0.748**	**0.783**	**0.900**	**0.796**	**0.759**	**0.850**	**0.787**
IpF3H	0.002	0.001		**0.698**	**0.823**	**0.965**	**0.761**	**0.878**	**0.914**	**0.794**
IpF3'H	0.002	0.041	0.018		0.385	**0.684**	**0.888**	0.359	**0.686**	**0.786**
IpDFR-B	0.080	0.088	0.097	0.103		**0.854**	0.477	**0.849**	**0.799**	**0.597**
IpANS	0.036	0.025	0.008	0.016	0.077		**0.773**	**0.832**	**0.932**	**0.746**
Ip3GT	0.090	0.008	0.096	0.002	0.195	0.057		0.466	**0.727**	**0.766**
IpMYB1	0.029	0.006	0.023	0.002	0.086	0.026	0.086		**0.749**	**0.598**
IpbHLH2	0.031	0.005	0.013	0.048	0.027	0.010	0.114	0.004		**0.777**
IpWDR1	0.087	0.048	0.052	0.079	0.024	0.050	0.051	0.153	0.040	

The upper triangle shows the means over two replicates while the lower one shows the standard errors. A total of 31 developmental stages of petals were sampled. Correction for multiple comparisons (k=45) used Dunn-Šidák method, at the experiment-wise error rate $\alpha = 0.05$; individual *t*-test with the P-value smaller than 0.001 was shown in bold.

Supplementary Table S4. List of 159 sequences of the anthocyanin genes at the 5' non-coding region.

**Coding ID**	**5' Non-coding Region**	**Species**	**Reference**
>AAF19756.1	nucl:CP002684.1 <10812918..10814917>	*Arabidopsis thaliana*	*3GT_b*
>CAC01717.1	nucl:CP002688.1 <5607476.. 5609475>	*Arabidopsis thaliana*	*3GT_b*
>AAS99853.1	nucl:AY585677.2 <327..2326>	*Allium cepa*	*ANS_c*
>ACC66092.1	nucl:EU600205.1 <1..620>	*Ginkgo biloba*	*ANS_c*
>BAB71810.1	nucl:AB073924.1 <1..1056>	*Ipomoea nil*	*ANS_c*
>BAB61138.1	nucl:AP003198.3 <71227.. 73226>	*Oryza sativa Japonica Group*	*ANS_c*
>BAD37752.1	nucl:AP004737.3 <124747.. 126746>	*Oryza sativa Japonica Group*	*ANS_c*
>BAE98273.1	nucl:AB247917.1 <109..2108>	*Triticum aestivum*	*ANS_c*
>BAE98275.1	nucl:AB247919.1 <1..1044>	*Triticum aestivum*	*ANS_c*
>BAE98277.1	nucl:AB247921.1 <1..1641>	*Triticum aestivum*	*ANS_c*
>CAN68377.1	nucl:AM473498.2 <4864..6863>	*Vitis vinifera*	*ANS_c*
>CAA39022.1	nucl:X55314.1 <1..1291>	*Zea mays*	*ANS_c*
>BAF46858.1	nucl:AB252661.1 <417..2416>	*Ipomoea purpurea*	*bHLH2_b*
>BAF46859.1	nucl:AB252663.1 <1746..3745>	*Ipomoea purpurea*	*bHLH2_b*
>BAD18984.1	nucl:AB154372.1 <1..922>	*Ipomoea tricolor*	*bHLH2_b*
>AAG25927.1	nucl:AF260918 <1..601>	*Petunia x hybrida*	*bHLH2_b*
>CAN62848.1	nucl:AM436565.2 <20116.. 22115>	*Vitis vinifera*	*bHLH2_b*
>CAB94968.1	nucl:AJ287322.1<1..234>	*Arabidopsis lyrata*	*CHI_fl1*
>AAA32766.1	nucl:ATHCFI <1..661>	*Arabidopsis thaliana*	*CHI_fl1*
>BAB10427.1	nucl:CP002688.1 <26459178..26461177>	*Arabidopsis thaliana*	*CHI_fl1*
>AAM13449.1	nucl:AF474923.1 <4938..6937>	*Hordeum vulgare subsp. vulgare*	*CHI_fl1*
>AAO65886.1	nucl:AC104433.8 <55507.. 57506>	*Oryza sativa Japonica Group*	*CHI_fl1*
>EU032606.1	nucl:KC794944.1<1..745>	*Ipomoea purpurea*	*CHI_fl2*
>CAA27338.1	nucl:AB691773.1 <1..1179>	*Antirrhinum majus*	*CHSD_us1*
>CAI30817.1	nucl:AJ868240.1<1..1362>	*Arabidopsis croatica*	*CHSD_us1*
>AAC24368.1	nucl:CP002684.1 <360441.. 362440>	*Arabidopsis thaliana*	*CHSD_us1*
>BAB11121.1	nucl:CP002688.1 <4486763..4488762>	*Arabidopsis thaliana*	*CHSD_us1*
>BAA03784.1	nucl:DARGCHS2 <431..2430>	*Daucus carota*	*CHSD_us1*
>BAF62128.1	nucl:AB480070.1 <2828..4827>	*Glycine max*	*CHSD_us1*
>AAQ62588.1	nucl:AY262686.1 <77643.. 79642>	*Glycine max*	*CHSD_us1*
>AAQ62595.1	nucl:EF623854.1 <42890..44889>	*Glycine max*	*CHSD_us1*
>AAQ62590.1	nucl:EF623856.1 <121254..123253>	*Glycine max*	*CHSD_us1*
>ABQ63059.1	nucl:EF623856.1 <127194..129065>	*Glycine max*	*CHSD_us1*
>AAA33950.1	nucl:EF623857.1 <48797.. 49594>	*Glycine max*	*CHSD_us1*
>AAO67373.1	nucl:EF623858.1 <129142.. 129977>	*Glycine max*	*CHSD_us1*
>AAA33951.1	nucl:SOYCHSVI <1..645>	*Glycine max*	*CHSD_us1*
>CAA41250.1	nucl:X58339.1 <8..2007>	*Hordeum vulgare*	*CHSD_us1*

>CAD23044.1	nucl:AJ430353.1 <1..1425>	*Humulus lupulus*	*CHSD_us1*
>BAA87336.1	nucl:AB027533.1<1..896>	*Ipomoea nil*	*CHSD_us1*
>BAA87338.1	nucl:AB027535.1 <1..1747>	*Ipomoea nil*	*CHSD_us1*
>AAK39113.1	nucl:AF358657 <1..2000>	*Ipomoea purpurea*	*CHSD_us1*
>AAK39115.1	nucl:AF358659 <2973..4972>	*Ipomoea purpurea*	*CHSD_us1*
>ABF82595.1	nucl:DQ471951.1 <1..897>	*Lilium hybrid cultivar*	*CHSD_us1*
>AAL77133.1	nucl:AC098566.3 <133847.. 135846>	*Oryza sativa*	*CHSD_us1*
>BAF26111.1	nucl:AC116600.1 <22289..24288>	*Oryza sativa Japonica Group*	*CHSD_us1*
>BAF26141.1	nucl:AC131375.1 <97154..99153>	*Oryza sativa Japonica Group*	*CHSD_us1*
>BAF28472.1	nucl:AC133291.2 <91346..93345>	*Oryza sativa Japonica Group*	*CHSD_us1*
>BAF28372.1	nucl:AC134256.4 <29294.. 31293>	*Oryza sativa Japonica Group*	*CHSD_us1*
>BAF28370.1	nucl:AC135568.3 <147817..149816>	*Oryza sativa Japonica Group*	*CHSD_us1*
>BAF28371.1	nucl:AC135568.3 <158232.. 160231>	*Oryza sativa Japonica Group*	*CHSD_us1*
>AAT44239.1	nucl:AC136226.2 <23527.. 25526>	*Oryza sativa Japonica Group*	*CHSD_us1*
>BAF16836.1	nucl:AC136226.2 <33660.. 35659>	*Oryza sativa Japonica Group*	*CHSD_us1*
>BAF13917.1	nucl:AL662935.3 <49127.. 51126>	*Oryza sativa Japonica Group*	*CHSD_us1*
>CAE05340.2	nucl:AL731609.2 <137936.. 139935>	*Oryza sativa Japonica Group*	*CHSD_us1*
>BAD30758.1	nucl:AP004573.3 <112169.. 114168>	*Oryza sativa Japonica Group*	*CHSD_us1*
>BAF21742.1	nucl:AP004573.3 <76837.. 78836>	*Oryza sativa Japonica Group*	*CHSD_us1*
>BAF21744.1	nucl:AP005169.3 <33839.. 35838>	*Oryza sativa Japonica Group*	*CHSD_us1*
>BAD31062.1	nucl:AP005177.4 <135141.. 137140>	*Oryza sativa Japonica Group*	*CHSD_us1*
>CAA32731.1	nucl:X14591.1 <1..1226>	*Petunia x hybrida*	*CHSD_us1*
>CAA32737.1	nucl:X14597.1 <1..1468>	*Petunia x hybrida*	*CHSD_us1*
>AAV70116.1	nucl:AY825502.1 <1..619>	*Phalaenopsis hybrid cultivar*	*CHSD_us1*
>BAA22042.1	nucl:D88260.1 <1..404>	*Pisum sativum*	*CHSD_us1*
>BAA22043.1	nucl:D88261.1 <1..842>	*Pisum sativum*	*CHSD_us1*
>BAA01512.1	nucl:PEACHS1 <1..1571>	*Pisum sativum*	*CHSD_us1*
>CAA56316.1	nucl:X80007.1 <1..1040>	*Pisum sativum*	*CHSD_us1*
>BAA87925.1	nucl:AB022685.1 <1..241>	*Psilotum nudum*	*CHSD_us1*
>ABV54603.1	nucl:EF694718.1 <1..230>	*Rubus idaeus*	*CHSD_us1*
>ACF72868.1	nucl:EU862821.1 <1..1194>	*Rubus idaeus*	*CHSD_us1*
>CAA63306.1	nucl:X92548.1 <1..1041>	*Secale cereale*	*CHSD_us1*
>AAD41873.1	nucl:AF152548 <1..519>	*Sorghum bicolor*	*CHSD_us1*
>AAD41874.1	nucl:AF152549 <1..581>	*Sorghum bicolor*	*CHSD_us1*
>AAD41877.1	nucl:AF152552 <1..461>	*Sorghum bicolor*	*CHSD_us1*
>AAA73939.1	nucl:TFRCHS5AAA <1..601>	*Trifolium subterraneum*	*CHSD_us1*
>AAA67701.1	nucl:TFRCHS6A <1..601>	*Trifolium subterraneum*	*CHSD_us1*
>CAN82080.1	nucl:AM424663.2 <15459.. 17458>	*Vitis vinifera*	*CHSD_us1*
>CAN62494.1	nucl:AM427145.2 <716..2715>	*Vitis vinifera*	*CHSD_us1*
>CAN82569.1	nucl:AM434769.2 <4798.. 6797>	*Vitis vinifera*	*CHSD_us1*
>CAN76172.1	nucl:AM439846.2 <8224.. 10223>	*Vitis vinifera*	*CHSD_us1*
>CAN61105.1	nucl:AM450362.2 <4434.. 6433>	*Vitis vinifera*	*CHSD_us1*

>CAN72348.1	nucl:AM459972.2 <18080.. 20079>	*Vitis vinifera*	*CHSD_us1*
>CAN68070.1	nucl:AM463938.2 <16979.. 18978>	*Vitis vinifera*	*CHSD_us1*
>CAN75038.1	nucl:AM472295.2 <3027.. 4339>	*Vitis vinifera*	*CHSD_us1*
>CAN81926.1	nucl:AM485293.2 <4641.. 6640>	*Vitis vinifera*	*CHSD_us1*
>CAN81964.1	nucl:AM487161.2 <7741.. 9740>	*Vitis vinifera*	*CHSD_us1*
>AAW56961.1	nucl:AY728476.1 <7778.. 9777>	*Zea mays*	*CHSD_us1*
>AAW56964.1	nucl:AY728478.1 <721..2720>	*Zea mays*	*CHSD_us1*
>ACN31816.1	nucl:BT065940.1 <1..645>	*Zea mays*	*CHSD_us1*
>CAA42763.1	nucl:X60204.1 <1..512>	*Zea mays*	*CHSD_us1*
>BAH36919.1	nucl:AB276104.1<1..369>	*Aegilops longissima*	*DFRB_fl2*
>BAH36921.1	nucl:AB276105.1<1..370>	*Aegilops sharonensis*	*DFRB_fl2*
>BAE78769.1	nucl:AB099529.1 <1..664>	*Agapanthus praecox*	*DFRB_fl2*
>AAO63026.1	nucl:AY221250.2 <699..2698>	*Allium cepa*	*DFRB_fl2*
>BAA85261.1	nucl:AB033294.1 <595..2594>	*Arabidopsis thaliana*	*DFRB_fl2*
>AAD21417.1	nucl:CP002684.1 <22792757.. 22794756>	*Arabidopsis thaliana*	*DFRB_fl2*
>AAS89833.1	nucl:AY575057.1 <1..1442>	*Fragaria x ananassa*	*DFRB_fl2*
>ABM64803.1	nucl:EF187612.1 <43..1478>	*Glycine max*	*DFRB_fl2*
>AAB20555.1	nucl:S69616.1 <1..1340>	*Hordeum vulgare*	*DFRB_fl2*
>AB112545	nucl:AB112545.1 <1..2689>	*Ipomoea batatas*	*DFRB_fl2*
>BAA22076.1	nucl:AB006793.1 <12376..14375>	*Ipomoea nil*	*DFRB_fl2*
>BAA59332.1	nucl:AB006793.1 <175..2174>	*Ipomoea nil*	*DFRB_fl2*
>ab006793	nucl:AB006793.1 <4801..9146>	*Ipomoea nil*	*DFRB_fl2*
>BAA36405.1	nucl:AB011667.1<1..1230>	*Ipomoea purpurea*	*DFRB_fl2*
>BAF64709.1	nucl:AB267077.1 <2140..4139>	*Ipomoea tricolor*	*DFRB_fl2*
>ABS84871.1	nucl:EF517133.1 <1..502>	*Linaria sp. JA-2007*	*DFRB_fl2*
>AAD26204.1	nucl:AF117268 <1..360>	*Malus x domestica*	*DFRB_fl2*
>CAH68068.1	nucl:AL732336.3<15176..17175>	*Oryza sativa Indica Group*	*DFRB_fl2*
>AAB58474.1	nucl:OSU70541 <24911..26910>	*Oryza sativa Indica Group*	*DFRB_fl2*
>BAF15893.1	nucl:AL606650.5 <59608..61607>	*Oryza sativa Japonica Group*	*DFRB_fl2*
>CAA56160.1	nucl:X79723.1 <1..1903>	*Petunia x hybrida*	*DFRB_fl2*
>AAB94015.1	nucl:AF010283.1 <37522..39521>	*Sorghum bicolor*	*DFRB_fl2*
>BAD11017.1	nucl:AB162138.1<1..491>	*Triticum aestivum*	*DFRB_fl2*
>BAH36899.1	nucl:AB276083.1<1..417>	*Triticum turgidum subsp. durum*	*DFRB_fl2*
>CAN76937.1	nucl:AM461096.2 <15451.. 17450>	*Vitis vinifera*	*DFRB_fl2*
>CAA75996.1	nucl:Y16040.1 <1..678>	*Zea mays*	*DFRB_fl2*
>CAB62646.1	nucl:CP002686.1 <19023410..19025409>	*Arabidopsis thaliana*	*F3H_fl1*
>CAA49839.1	nucl:X70378.1 <1..604>	*Dianthus caryophyllus*	*F3H_fl1*
>AAU04792.1	nucl:AY691919.1 <1..446>	*Fragaria x ananassa*	*F3H_fl1*
> AAB41102.1	nucl:AB154372.1 <3074.. 3350>	*Ipomoea purpurea*	*F3H_fl1*
>BAF16071.1	nucl:AL606999.3 <57999.. 59998>	*Oryza sativa Japonica Group*	*F3H_fl1*
>BAH36892.1	nucl:AB223024.1 <1..735>	*Triticum aestivum*	*F3H_fl1*
>BAH36893.1	nucl:AB223025.1 <1..732>	*Triticum aestivum*	*F3H_fl1*

>BAH36894.1	nucl:AB223026.1 <1..505>	*Triticum aestivum*	*F3H_fl1*
>CAN80262.1	nucl:AM430949.2 <3835..5834>	*Vitis vinifera*	*F3H_fl1*
>CAN61951.1	nucl:AM477026.2 <7070.. 8429>	*Vitis vinifera*	*F3H_fl1*
>CAB45977.1	nucl:CP002687.1 <7309692.. 7311691>	*Arabidopsis thaliana*	*F3'H_purp*
>CAB62611.1	nucl:CP002688.1 <2558438..2560437>	*Arabidopsis thaliana*	*F3'H_purp*
>BAD00191.1	nucl:AB113266.1 <1..1308>	*Ipomoea purpurea*	*F3'H_purp*
>BAD00192.1	nucl:AB113268.1 <1..1194>	*Ipomoea tricolor*	*F3'H_purp*
>AAK92618.1	nucl:AC078944 <143299..145298>	*Oryza sativa Japonica Group*	*F3'H_purp*
>BAF26135.1	nucl:AC131375.1 <16233..18232>	*Oryza sativa Japonica Group*	*F3'H_purp*
>AAM00948.1	nucl:AC131968.1 <4533..6532>	*Oryza sativa Japonica Group*	*F3'H_purp*
>BAD23209.1	nucl:AP004022.3 <37975..39974>	*Oryza sativa Japonica Group*	*F3'H_purp*
>BAD38068.1	nucl:AP005419.3 <41891.. 43890>	*Oryza sativa Japonica Group*	*F3'H_purp*
>BAD10411.1	nucl:AP005483.3 <60681.. 62680>	*Oryza sativa Japonica Group*	*F3'H_purp*
>BAD36157.1	nucl:AP005570.3 <125088.. 127087>	*Oryza sativa Japonica Group*	*F3'H_purp*
>BAF24627.1	nucl:AP006446.3 <41891.. 43890>	*Oryza sativa Japonica Group*	*F3'H_purp*
>CAN80142.1	nucl:AM428246.2 <17818..19817>	*Vitis vinifera*	*F3'H_purp*
>CAN60359.1	nucl:AM429113.2 <12308..14307>	*Vitis vinifera*	*F3'H_purp*
>CAN82588.1	nucl:AM436340.2 <10263.. 12262>	*Vitis vinifera*	*F3'H_purp*
>CAN68303.1	nucl:AM471220.2 <1025..3024>	*Vitis vinifera*	*F3'H_purp*
>CAN75347.1	nucl:AM484178.2 <6349.. 8348>	*Vitis vinifera*	*F3'H_purp*
>CAN62275.1	nucl:AM488740.1 <4082.. 6081>	*Vitis vinifera*	*F3'H_purp*
>CAN77776.1	nucl:AM488980.2 <20385.. 22384>	*Vitis vinifera*	*F3'H_purp*
>X96784.1	nucl:X96784.1 <1.. 950>	*Nicotiana tabacum*	*F3'H_purp*
>ABM66367.1	nucl:EF192598.1 <1091..3090>	*Allium cepa*	*IpANS_c*
>EU032614.1	nucl:KC794942.1 <1..359>	*Ipomoea purpurea*	*IpANS_f*
>BAG68211.1	nucl:AB576766.1 <183..2182>	*Ipomoea batatas*	*MYB1*
>ab232773	nucl:AB232773.1 <1..2642>	*Ipomoea nil*	*MYB1*
>BAE94710.1	nucl:AB234212.1 <4398..6397>	*Ipomoea nil*	*MYB1*
>ACO69142.1	nucl:CP001575.1 <455396..457395>	*Micromonas sp. RCC299*	*WDR1_a*
>BAF09665.1	nucl:AP004178.3 <55138.. 57137>	*Oryza sativa Japonica Group*	*WDR1_a*
>BAF08903.1	nucl:AP004772.3 <97485.. 99484>	*Oryza sativa Japonica Group*	*WDR1_a*
>CAB69198.1	nucl:AJ133743.1 <2401..2940>	*unidentified*	*WDR1_a*
>CAN66600.1	nucl:AM423597.2 <1368..3367>	*Vitis vinifera*	*WDR1_a*
>AAM76742.1	nucl:AY115485.1 <1321..3320>	*Zea mays*	*WDR1_a*
>AAR01949.1	nucl:AY339884.1 <1..1445>	*Zea mays*	*WDR1_a*
>CAB45372.1	nucl:AJ133743.1 <1382..3381>	*Arabidopsis thaliana*	*WDR1a*
>BAE94401.1	nucl:AB232783.1<3076..5075>	*Ipomoea nil*	*WDR1a*
>AB232777	nucl:KC794945.1<1..2000>	*Ipomoea purpurea*	*WDR1a*

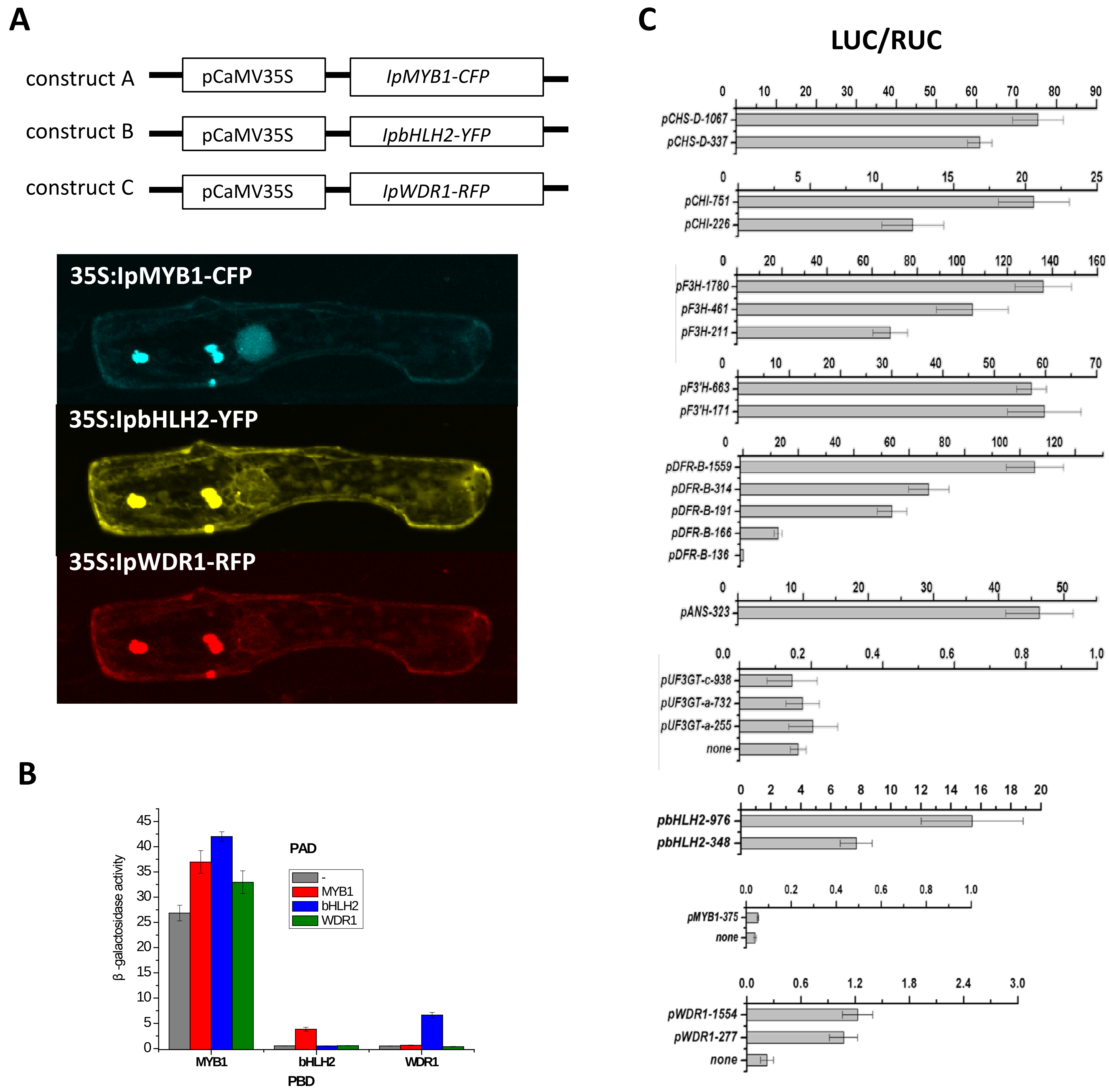

Supplementary Fig. S1. Characterization of *Ipomoea* TFs and promoter activities of the target genes.

(A) Subcellular locations of IpMYB1, IpbHLH2, and IpWDR1. Three constructs were used in the transient expressions as shown, which were introduced into onion epidermal cells by particle bombardment. The white spots are artifacts. (B) TF-TF interactions in the yeast two-hybrid experiments. The results show *β*-galactosidase activities of different combinations of protein interactions. TFs in (A) were integrated into both pBD and pAD vectors, and pairs of TF vectors co-transformed yeast strain YRG-2. The error bars were based on six independent tests. Empty vectors were shown in "-". (C) The promoter deletion trials of anthocyanin genes. Dual luciferase assays were conducted in *Ipomoea nil wdr1* mutant (strain baichou). The promoter activity was measured in LUC/RUC with at least three replicates (errors shown in bars) per test.

1	BRE-m4b	TTCACTTGTTTTCACTTGTTTTCACTTGTTTTCACTTGTT
2	BRE-m5b	TTCACGGGTTTTCACGGGTTTTCACGGGTTTTCACGGGTT
3	BRE-m6b	TTCACGTCTTTTCACGTCTTTTCACGTCTTTTCACGTCTT
4	BRE-m1	TTCACCTGTTTTCACCTGTTTTCACCTGTTTTCACCTGTT
5	F3’H-BRElike	TTGTGCATTTTTGTGCATTTTTGTGCATTTTTGTGCATTT
6	F3’H-BRE	TTCACCCGTTTTCACCCGTTTTCACCCGTTTTCACCCGTT
7	F3H-BRE	TTCACGTTTTTTCACGTTTTTTCACGTTTTTTCACGTTTT
8	3GT-BRElike	TTCACGACTTTTCACGACTTTTCACGACTTTTCACGACTT
9	MlCHS-BRE	CAAGACCACGTGGCTCTCATCTACCCGACCAT
10	Mlrandom-p1	GGTGCCCCAACAATCTCGGGTGCAGTATCCGG
11	Mlrandom-p2	TACCGCCACTGCCACCAGCGCTCGATGAACTGCCGTGCGT
12	Mlrandom-p3	TGATTGGTTTTGGAGGACCATGTGTTTGAGTGCT

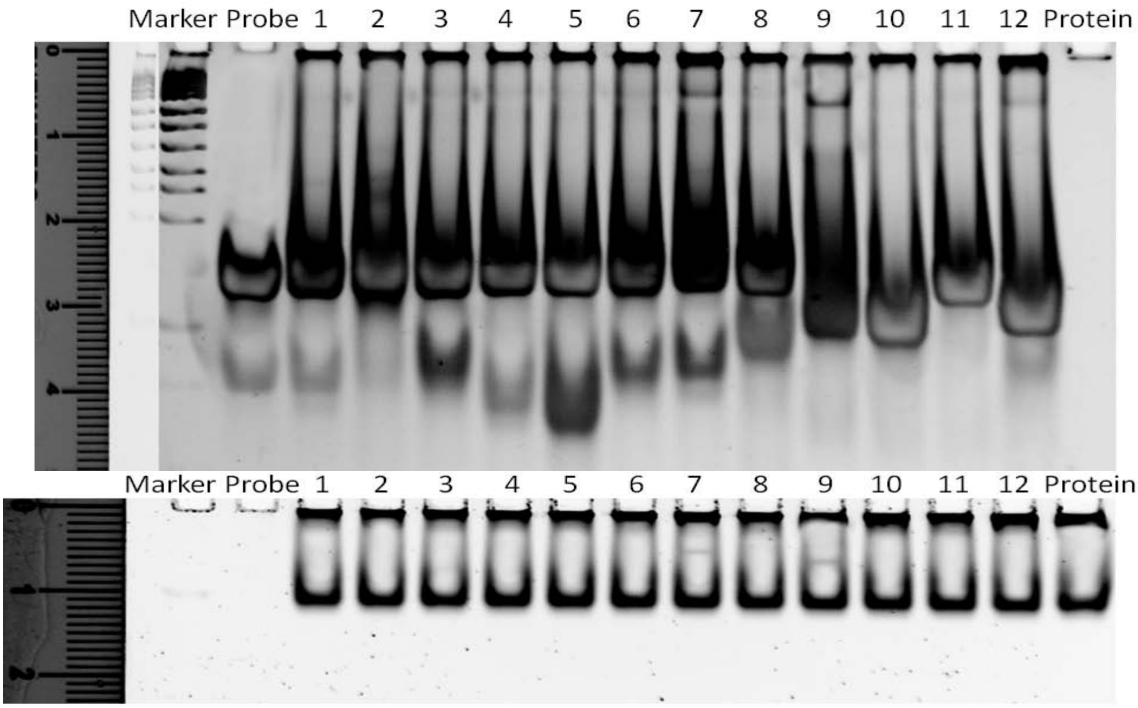

Supplementary Fig. S2. Binding capacity of IpbHLH2 in EMSAs. Numbered synthetic probes harbor variants of candidate BREs as underlined. DNA bindings are shown in the upper gel panel and the protein bindings of the same gel are in the lower panel. The lanes are labeled according to the probe numbers. The ruler is in the unit of cm, showing the position of the binding in each staining. The MBP-IpbHLH2 (protein) and the probe 1 (probe) were included as negative controls.

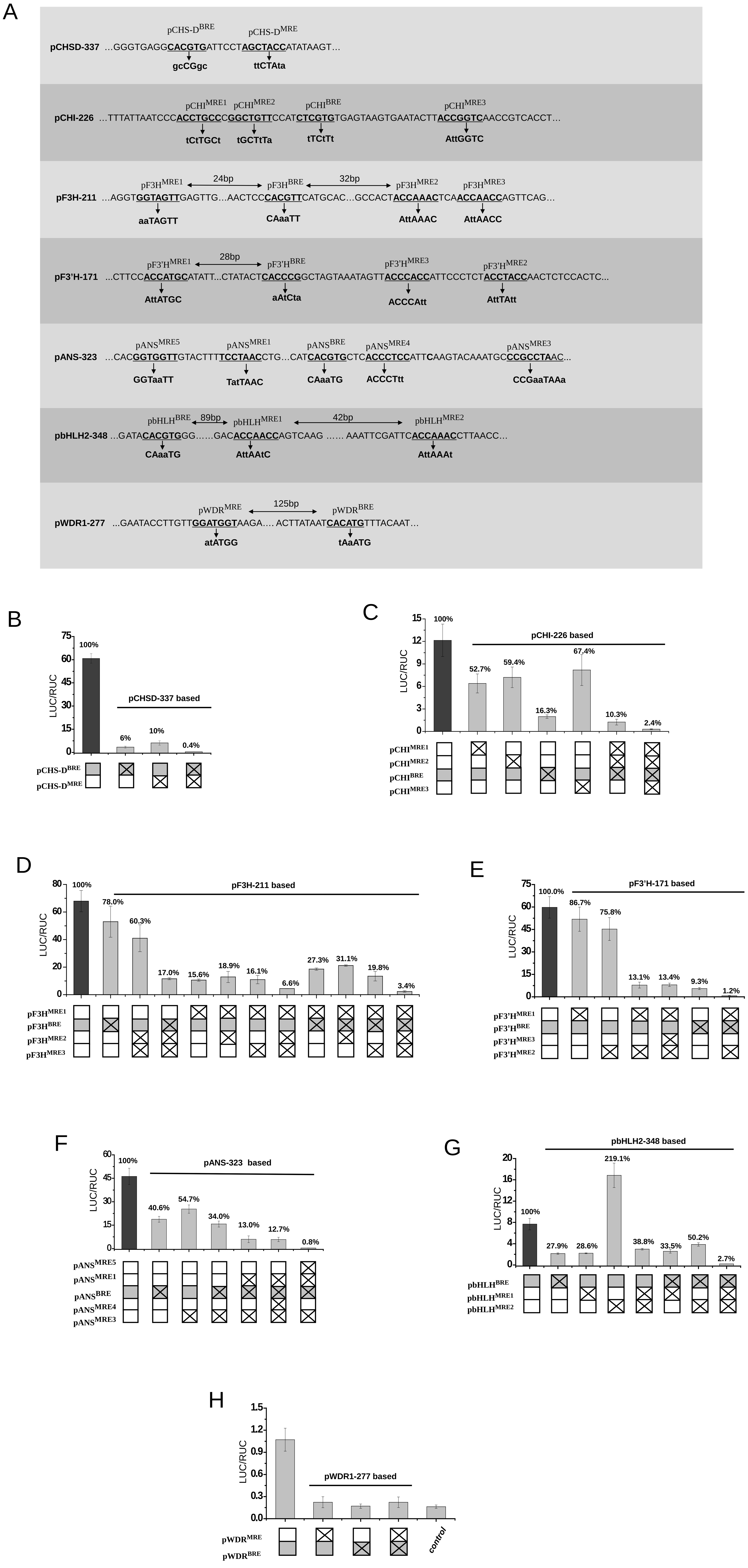

Supplementary Fig. S3. Analysis of *cis* elements on the anthocyanin promoters of *Ipomoea purpurea*.
(A) Sequence dissection of the proximal promoters (5'→3'), showing features of predicted *cis* motifs (bold and underlined) for IpMBW activation and the mutated sites (lower case). The labeled MREs and BREs are candidates only. (B)-(I) Results of dual luciferase assays for constructs hosting the promoter sequences of *CHS-D,CHI, F3H, F3'H, DFR-B, ANS, bHLH2*, and *WDR1*. The mutation types are indicated by checked boxes below the x-axis. The Y-axis shows the level of promoter activity in LUC/RUC. The original promoter activities are shown in black column and set as 100% in activity. The standard error bars are based on three replicates.

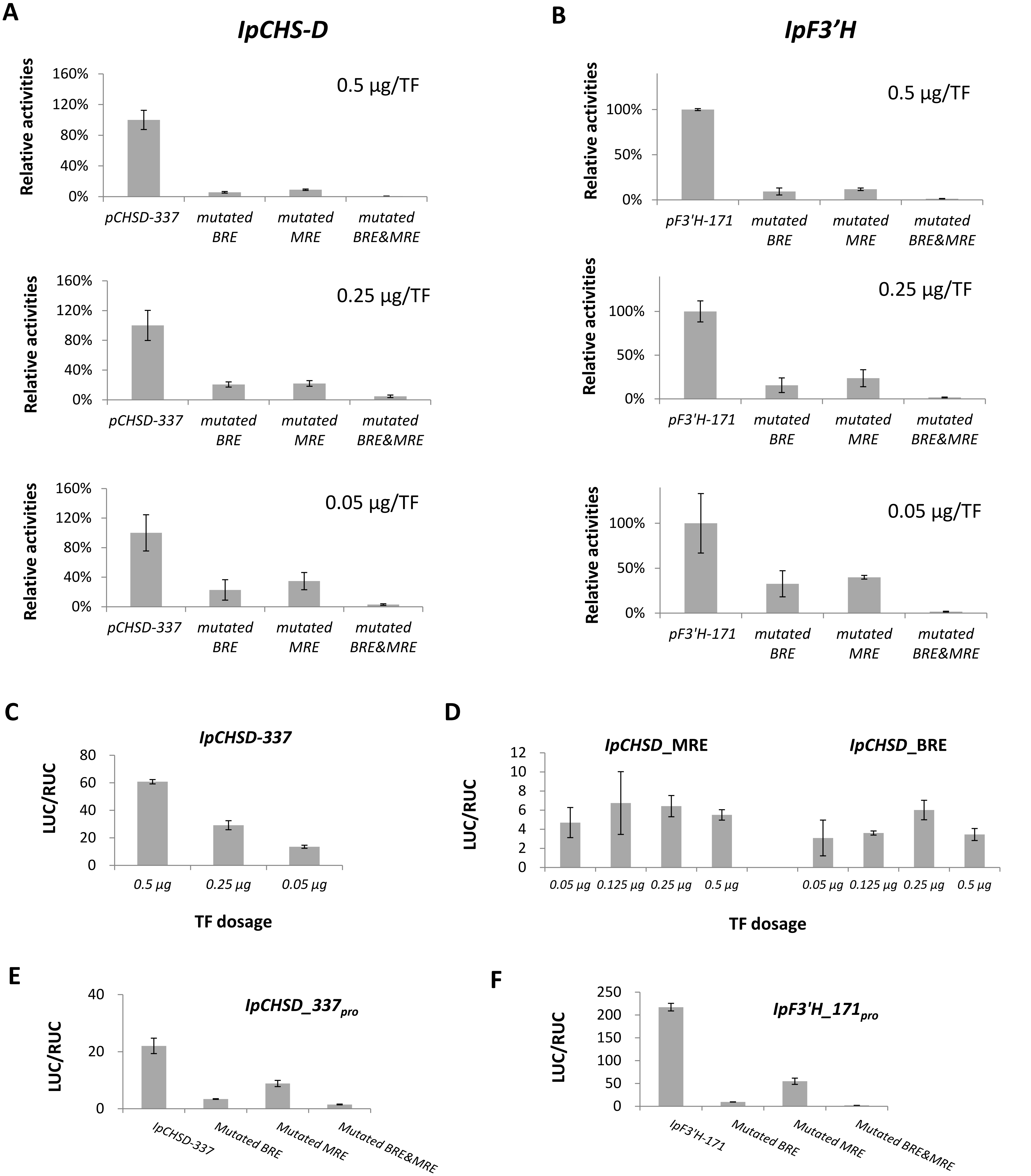

Supplementary Fig. S4. Comparisons of TF dosages in dual luciferase assays. (A) Dosage effect of IpMBW on *IpCHSD-337*$_{pro}$. Three levels of IpMBW were tested on the same amount of reporter (0.5 µg) in the transient expressions. Their effects on evaluations of mutations are expressed as percentages to the original *pCHSD-337* activity. (B) Dosage effect of IpMBW on *IpF3'H-171*$_{pro}$. The tests followed the same protocol as in (A). (C) Comparisons of the original *pCHSD-337* activities under three TF dosages. (D) Activities of *pCHSD-337* containing mutated MRE and BRE under different levels of IpMBW. The transient expressions were normalized across tests to show the transcription levels detected by LUC relative to RUC. (E) –(F) Transient expressions conducted in rice (cultivar Chujing 26) leaves. The procedures followed (A), except the TF dosage used 0.3:0.3:0.1µg for IpMYB1:IpbHLH2:IpWDR1. All error bars are from three replicates.

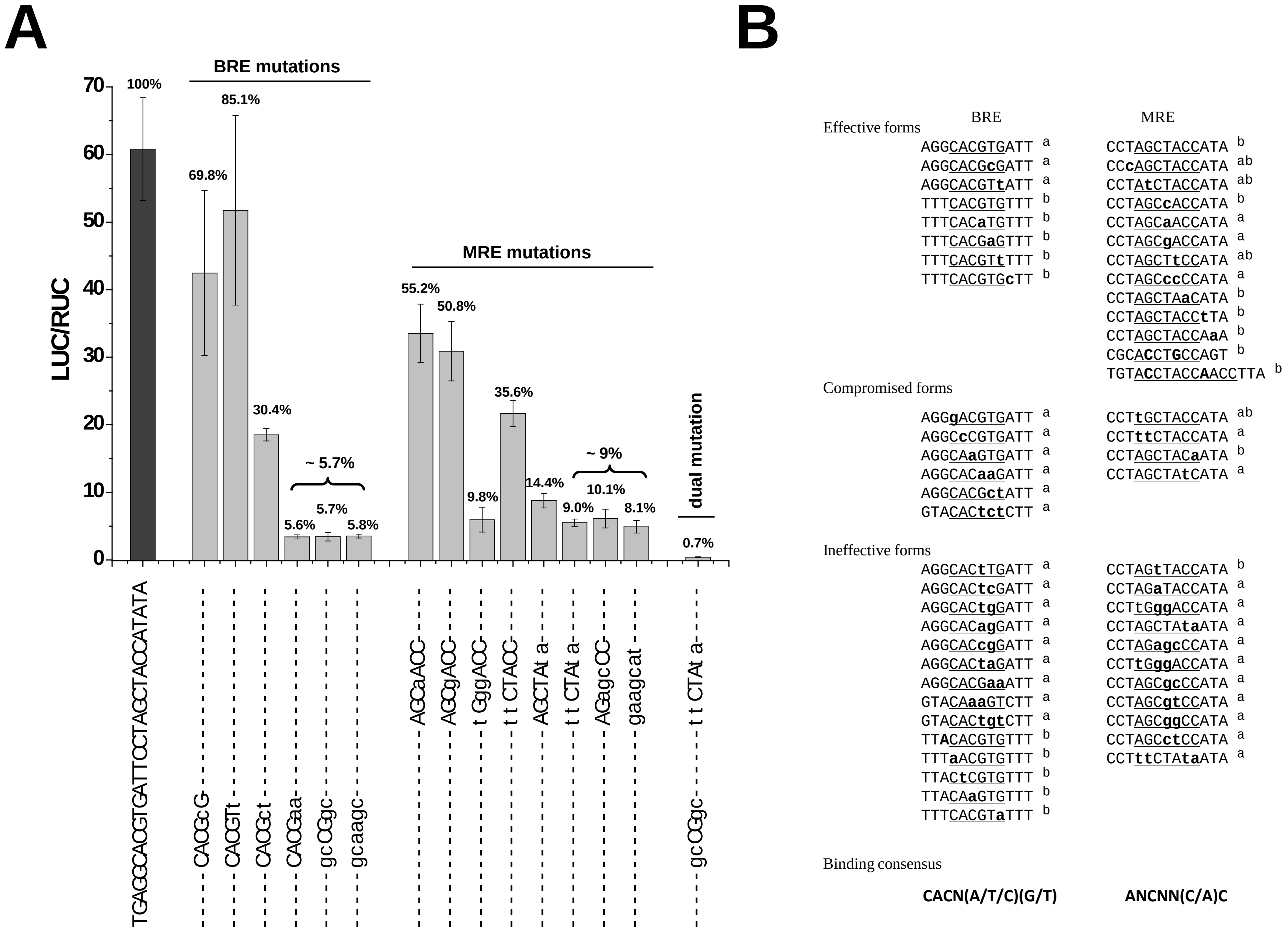

Supplementary Fig. S5. Effects of mutations introduced on *IpCHSD-337*$_{pro}$ showing broad DNA binding capacity of IpMYB1 and IpbHLH2. (A) Results of dual luciferase assays on mutated *IpCHSD-337* constructs driven by IpMYB1- IpbHLH2 - IpWDR1. The column for the wild type is in black and set as 100%. Activities of other mutant constructs relative to the wild type are indicated. The standard error is based on four independent trials. The sequences of the targeted series of constructs are shown along the x-axis. Mutated sites are shown by letters in lower case, and the wild type context by dashes. (B) Sequence summaries of MRE and BRE mutants reveal broad binding abilities of IpMYB1 and IpbHLH2. The consensus binding sites are mainly drawn from the effective binding sequences. The flanking nucleotides were also indicated. Sequence mutants not demonstrated in (A) are cited from Wang et al.(2013).

[a], data collected from dual luciferase assays.
[b], data collected from EMSAs.

Arabidopsis thaliana

>AtCHS-728

GTTGGAAAGCGCAAATAG**GGCAGAT**TTTCAGACAGATATCACTATGAT**GGGGGGT**GAGAGAAAGAAAACGAGGCGT
ACCTAATGTAACACTACTTAATTAGTCGTTAGTTATAGGACTTTTTTTTTGTTTGGGCCTAGTTATAGGATCATAA
GGTAAAAATGAAGAATGAATATTAGATTAGTAGGAGCTAATGATGGAGTTAAGTATG**CACGTG**TAAGAACTGGGAA
GTGAAACCTCCTGTATGGTGAAGAAACTATACAACAAAGCCCTTTGTTGGTGTATACGTATTAATTTTTATTCTTT
TATCACAAGCGATACGTATCTTAAGACATAATAAATATATATCTTACTCATAATAAATATCTTAAGATATATATAC
AGTATACACCTGTATATATATAATAAATAGGCATATAGTAGAAATTAATATGAGTTGTTGTTGTTGCAAATATATA
AATCAATCAAAAGATTTAAA**ACCCACC**ATTCAATCTTGGTAAGTAACGAAAAAAAAGGGAAGCAAGAAGAACCACA
GAAAAGGGGGCTAACAACTAGACACGTAGATCTTCATCTGCCCGTCCATCTA**ACCTACC**ACACTCTCATCTTCTTT
TTCCCGTGTCAGTTTGT***TATATAA***GCTCTCACTCTCCGGTATATTTCCAAATACACCTAACTTGTTTAGTACACAA
CAGCAACATCAAACTCTAATAAACCCAAGTTGGTGTATACTATA**ATG**

>AtCHI-217

GCTCAAAGTTTC**AACCACC**AATTGTCAATGCATCTCCCAATCAAAATATAAAAAAAGAGACGAAAACGAAAGTA**CA**
CGTGCTTACACATCCAACACTCGTAATCGTAACTATTGCT**ACCTACC**CTTCTCTCTTCTACTTAACCCCAAAGGCC
CCAAAAACACAATCAAGAAAAGCTCTGTAACATTATTATCAATCGAAATTCCAACCGACTCAACA**ATG**

>AtF3H-495

CGCAAGCCCGTACCAGAACATGTCTCCGC**CACGTG**ATTTCTCCACAGACCACAAGCATTTTTAAGACGTGGCTTTC
TATCAACCGTTAAAAACGTAAATCATATTAAC**CATGTG**TCTACTACCTACGGTGTAAACGAAACTGTATAACGTCC
CTATCATATAATAGTAATGTGATACGTTGGAATGTAGCCAAAAAGCATAAAAAATAAATAGATAATTAAGTTTATA
ATGTTTTCCTACAAAATATTATTATACCGTATGTATTTTTTATTTTATTTTCTGAAGTTAAAAACAGATGTAGTTA
GTTGAGTAAATTGTGTTCTAGAAAGAGAAGAGAGAGCAGTAGTACCGT**GGTAGGT**AGCTAGCGACCTCTTCGTTCG
TCAGTCATCACAAGCTTTGAAAGATTTTCAGCTACCACTCTCTCCTT***TATATAT***TCATTACACATCTCTTCTTTCT
ATATCTCTCTTAATTTAGTCTTTTGTCTTCGTAATTACA**ATG**

>AtF3'H-876

AAGGT***ACCAAAC***TAATGAACTGTAACCTCTTTTTTCTTTTCTTTTTTGTTAAAGGATTTATGAACTGTAACTTAGA
ATGCTTGGTTTGTGGGCAGTGTAATATATGACA**CACATG**CATTTTTTTTGTTTGTCAAATAGGAAGACTTCTTTTT
TCTTTATCAACTTCCTTATTTTCATAAAACAAAACACTGAAAAAAGTACAGATGTTCTCACGTACGT**CACGTG**TAC
ATACATATATATTAGACCACTATATAATAAGATATGAAGTGTTAGGTTTAAATCAATTAACGAATCCCATCCAAAT
GATGAAACAGTTAACAAGAAATCAAAATAGTTTATTAGGGTTACAATGATTTTATACTTTTAAGAAATCTTAGAAC
CTATCACTTACAAATGAGTAAATGACCATTACTCCTCGAGAATCTAAGGCGCTTAAGGAAGCATTGCGAATCGGGT
GTGAAAAAGATCTATTTTTGAATTATTTCACACAATTTCTTAATGTCAATTTTCGATGCTCCCATATTCTCCACGG
TTTAAAGCAAGATTGGTGGGAAAGGGATATTCTCGCATCGATTACAATGAAATATGGGTTGAAAAAAAATAAAAAA
ATTACTCAATGTTGCACCAAAAACCAGAAAACTCTAAGTTGCGCTAATAAAAAAAAAAGTTATAAACCCAACATCA
AACCAAAACCGTACTAAACTGTCCCATATGAGATTTAGCTTTAAATAAATTAGTACTTCTCATAACGATAACTAAA
TTAAATTTCCCTAGCCAAGACATACATATAGTTTTGATTGACAAAAAAAAAAAAACTCCTC***TATTTAT***AGCTTGTG
TTTTGTTTCCTCATTTTTCACTTACCATTCAAACCCAACACT**ATG**

>AtDFR-343

CTGAACTGAAGTCACCCACACGTCTC***ACCAAAC***AAATCGAAGTCAACGTATTTC**ACCCACC**GGTACAACAACAAAA

TACACACCTAAGGAAATAATAAAATCAACTTACCAGATTGTTACGTACCACACATCTCTTTAGTCCTTCGTCAACC
AACGTTCCC**CACGTG**CTTCTCC**GGTTGGT**ACT**CACGTG**ACCGGCAGCTTCTCGTTCTTATTATCTGTTTTCTTCAA
TAACGATTCATAATCTCTAGTGTCT***TATTTAT***AATGTCTTCACATCACAAAGATTTGTACCGAACATACATAGTTG
AATCTTTCCCAAAGCACAATCTATCATATAACCACAAAA**ATG**

>AtANS-503

TCCAAACTACAAAAAGAAAATGTGGTTAGTAGAAGAACTAGTAGAGGTGAA**CACGTG**GAGACACGCTTAAAG**CACG**
CGACGAAGAA**CACGTT**GATAGCGATTATGGGTTTAATTCTATTGGGCCTTTTCTGGGAGTCTAGACCCAAGCCCAT
ATAGTAGTAATCTTTTTGACCAATCAGTCAACCCAACCATCCTCTCCCGTTGACCGTGAAGTGAGTCACGCACTTA
CCTCACAACAATAGCACTAACCACC**GGTAGCT**CTACAATGTCTCTTAGTTCGGTAACAAACTCTTCTAACTAAAAG
TATAGTAAAAACTTTGC***TATATAA***GAAAGAGTCTTTGCACATTTCATTTACTTGCAACCAATTACAAAAAAGAGTG
TAAGAAGAAAAACAAAACAAATCCATTTTTTTTATTACTCTGTTTTTTCCCCTGTTTTTAAGTTTATTTACTTCTT
ACTCTGTTTTCTGCTCTGTTTTAGCTTTAAACAGAAGACTAAAGAAG**ATG**

>At3GT-344

AGCGTAACGAACAAATAACGACTTTTAAATAGAGTCTATTAATTTGCTTAAAAAGGGGGCCGAATATTTCAGGCCA
TCAA**C**TTAC**CACGTG**AAGTCTGGTTTTGCTTTTGTTGATGTTACGATACCAT**CACGTG**TTTGCACTTACCAGGAGG
TTACACTTGCCCGGTCATTTGGTTACCCAATAACCACACACCATTCATCAGGACACTC*ACCAATC*TGATCCGTTAA
AGGTTTATG***TATTTAT***ACACATCCAACACACAACTTAGAAATTAACAAAAGAGAACAAAAAAAGTCTAATAAGGAT
CTTCCGGTAGTTAAAGTGAAATACAAGAGAGTCCAAGAAA**ATG**

>AtBAN-667

GAATGCTATTGCCAATGCCTTCTTTTGTTTTCGATTTAGGATTTACCCTCTCTTTTTTTTGTCTTCTTCACTTTTT
ATCTTTCAATGTAACTTTCTGGTTATTTTATCTTTGTTAAACTCTGTTATGGATTTGTAGCTTAAATATGATAAAA
TTGCTTAAGGCCAGATTCTGTGAAACATGGACAAGAACAGAGCAAGTTATGTTGAATTGACTCGTGTAATTCGTGA
AACAGAACATAGCAAGTCCAAGTTGTGTTAAAAACTGCAGAGAATTTGACAGA**TTGGTGG**AAGTAAAAAGCATTCT
TTTGCAACTCATTTTAAGATCGGCAAAGAAAAAATTGAAGTAACAGAACCTTACTGTAACACTATTCGTTACTCTA
AAGCTGTGTTATATTGTTTAGAGACAGAAATAATCAAACTCTTGTGATAATTT**GGTAGAT**GATAACAAATCAGAAC
TCTGAAGGTCAATCTTTTTTGATTCTTAGGTGAAGACAAGTTGGTTATTTCAAAGAT**CACGTG**CTTACCTTCTAAA
ACAGCCTTATTGATCTACTGTTGTACCTAATGAGCAAGGACTATTTGCAAATCTTTTTACTTCT***TATATAG***AAGTC
TCAAGACGATAAACTCATAACAACTAAATCTCTATCTCTGTAATTTCAAAAGTACAATC**ATG**

Ipomoea purpurea

>IpMYB1-375

GAGACTCGTGGCAGACAAGATTTTGTCAAATTAACTCCTCACGACTTGGATGTTAATGTTTTCAACTTTACATTAT
AAACGTCATAAACATTATAATTAAAATATAAAGGTTACTTTTTTTCTTTCTTCTCCGGGAAAGTAAACAAACTCTT
TTCCAAGAAAATAGACAAACTCAATCAATATTTCTGGGGATTACGTAATATTTTTTTGGT***TATATATT***ACAGTTCT
GGTAACTATGTCCTAGTGCTGTGTTCTATTCCACTTGTCGAAGTTTCTGTTTTCATTTTTCTGCATTTTGGCTCTA
GCTAGCTAGCTACCCACTGCACGCAAAACAACGTAAGTACCCACTACGTAAGCAATTTACGTCAGCCTGCC**ATG**

>Ip3GT- 255

TTCATAGTAACCAAAAGAGAAAATTTCAATTTTGGTCACACGACTATAGGGGCTTTGTTAATTTCAGCACCTAACT
TTCAAAAGTATCAATTTAGTACCTTGAGTATGTAATTTTTCTCAATTTTAGTCATTCCGGCCAATTTACTGATCAA

CTTCCACCGGAAAAATTAATAGGCACAAAACTAAGGGGCAAATAAGTCTTTTCCTGAGCACAAAACTTGTGTCCAG
CCGGAAAACCATTTCTCGCCGGAAAAA**ATG**

Supplementary Fig. S6. The 5' non-coding sequences of flavonoid genes of two species (*A. thaliana* and *I. purpurea*). All genes of *A. thalianan* are from the Columbia accession, and *IpMYB1-375* precedes the coding region of *IpMYB1* (AB232769), and *Ip3GT-255* is from *fl1* allele of *Ip3GT* (AF028237). Motifs conformed to the known syntaxes are colored (suspected MREs in red and suspected BREs in blue), and weak motifs in italic. The translation start site is in black, and the suspected TATA box in black and italic. BRE and MRE in proximity to TATA are in shade.

Supplementary File 1. Bioinformatic Survey and Perl Scripts.

(1) **Surveying promoters**

Script **WxW_Align.Gene** picks qualified promoters from PrmtrFasta, which is the file containing all promoter sequences in fasta format.

It reads each promoter sequence, and uses parameter Word1 (length of sliding window) to move from 5' to 3' to see if four or more than four of nucleotides satisfy CACNNG. When the condition hits, it will switch to Word2 (length of sliding window) to search from the current site till 100 bp to see if the second condition (>= 4 identical sites of ANCNNCC) meets. When both hits return, it will record the sequence in Selected to specify the pattern of Word1~Gap~Word2.

Parameters Word1TH and Word2TH are the smallest threshold values for Word1 and Word2, respectively.

usage: WxW_Align.Gene PrmtrFasta Word1 Word1TH Word2 Word2TH Selected

(2) **Counting the frequencies of *cis* pairs**

Script WxW_Align.PosD takes the Gap recorded in Selected to compute the frequency of each pattern of Word1~Gap~Word2 and writes it to PosD.

usage: WxW_Align.PosD PrmtrFasta Word1 Word1TH Word2 Word2TH PosD

PrmtrFasta was selected file in fasta；Word1 and Word2 follow the same notations above. PosD is output file recoding the frequency corresponding to Gap.

```
**********perl script： WxW_Align.Gene*****************
#!/usr/bin/perl -w
use strict;
my ($W1L,$W2L,%Prmtr,$L,$ID,$i,$j,$Qry,$Target,%Selected);

sub Align{
	my ($Qry,$Sbjt)=@_;
	my ($i,$Score,@Sbjt,@Qry);
	@Sbjt=split(//,$Sbjt); @Qry=split(//,$Qry);
	$Score=0;
	for($i=0;$i<@Sbjt;$i++){if($Sbjt[$i] eq $Qry[$i]){$Score++;}}
	return $Score;
}

sub Read{
	print "Reading ......\n";
	open(IN,"<$ARGV[0]") or die "Can't open $ARGV[0]!\n";
	while(<IN>){
		chomp;
		if($_=~/^>/){$ID=$_;}
		else{$Prmtr{$ID}=lc($_);}
	}
	close(IN);
}

sub Print{
	for $ID (keys %Prmtr){
		$L=length($Prmtr{$ID});
		for($i=0;$i<$L-100;$i++){
			$Qry=substr($Prmtr{$ID},$i,$W1L);
			if(Align($Qry,$ARGV[1])>=$ARGV[2]){
				$Target=uc($Qry); $Prmtr{$ID}=~s/$Qry/$Target/g;
				for($j=$i+$W1L+1;$j<$L-$W2L;$j++){
					$Qry=substr($Prmtr{$ID},$j,$W2L);
					if(Align($Qry,$ARGV[3])>=$ARGV[4]){
						$Target=uc($Qry); $Prmtr{$ID}=~s/$Qry/$Target/g;
						$Selected{$ID}++;
					}
				}
			}
		}
	}

	open(OUT,">$ARGV[5]") or die "Can't open $ARGV[5]!\n";
```

```
	for $ID (keys %Selected){print OUT "$ID\n$Prmtr{$ID}\n";}
	close(OUT);
}

# main program
@ARGV or die "Usage:$0 PrmtrInSimpleFasta Word1 Word1TH Word2 Word2TH Selected\n";
$W1L=length($ARGV[1]); $W2L=length($ARGV[3]);
Read();
Print();
```

**********perl script：WxW_Align.PosD*****************

```
#!/usr/bin/perl -w
use strict;
my ($W1L,$W2L,%Prmtr,$L,$ID,%PosD,$i,$j,$Qry1,$Qry2,$d);

sub Align{
	my ($Qry,$Sbjt)=@_;
	my ($i,$Score,@Sbjt,@Qry);
	@Sbjt=split(//,$Sbjt); @Qry=split(//,$Qry);
	$Score=0;
	for($i=0;$i<@Sbjt;$i++){if($Sbjt[$i] eq $Qry[$i]){$Score++;}}
	return $Score;
}

sub Read{
	print "Reading ......\n";
	open(IN,"<$ARGV[0]") or die "Can't open $ARGV[0]!\n";
	while(<IN>){
		chomp;
		if($_=~/^>/){$ID=$_;}
		else{$Prmtr{$ID}=lc($_);}
	}
	close(IN);
}

sub Parse{
	print "Parsing ......\n";
	$W1L=length($ARGV[1]); $W2L=length($ARGV[3]);
	for $ID (keys %Prmtr){
		$L=length($Prmtr{$ID});
		for($i=0;$i<$L-100;$i++){
			$Qry1=substr($Prmtr{$ID},$i,$W1L);
			if(Align($Qry1,$ARGV[1])>=$ARGV[2]){
				for($j=$i+$W1L;$j<$L-$W2L;$j++){
					$Qry2=substr($Prmtr{$ID},$j,$W2L);
					if(Align($Qry2,$ARGV[3])>=$ARGV[4]){
						$d=$j-$i-$W1L;
						$PosD{$d}++;
					}
				}
			}
		}
	}
```

```
sub Print{
	print "Printing ......\n";
	open(OUT,">$ARGV[5]") or die "Can't open $ARGV[5]!\n";
	for($i=0;$i<=100;$i++){
		if(defined $PosD{$i}){print OUT "$i\t$PosD{$i}\n";}
	}
	close(OUT);
}

# main program
@ARGV or die "Usage:$0 PrmtrFasta Word1 Word1TH Word2 Word2TH PosD\n";
Read();
Parse();
Print();
```
